# Functional Nanostructured Carbon Honeycomb Monoliths for Hemoadsorption: Preliminary Studies on Biocompatibility, Protein-Bound Uremic Toxins and Inflammatory Cytokines Elimination

**DOI:** 10.3390/ijms27177972

**Published:** 2026-09-07

**Authors:** Jakpar Jandosov, Carol Howell, Susan Sandeman, Dmitriy Chenchik, Sergey Mikhalovsky, Aitugan Sabitov, Joaquin Silvestre-Albero, Zulkhair Mansurov, Seitkhan Azat, Rosa Busquets, Nurzhamal Zhylybayeva, Mikhail Tsukerman, Alzhan Baimenov

**Affiliations:** 1 Institute of Combustion Problems, 172, Bogenbay Batyr St., Almaty 050000, Kazakhstan; aitugans@mail.ru (A.S.);; 2 Faculty of Chemistry and Chemical Technology, Al-Farabi Kazakh National University, 71, Al-Farabi Avenue, Almaty 050012, Kazakhstan; 3 Enteromed Ltd., 85 Great Portland St., London W1W 7LT, UK; 4 Centre for Regenerative Medicine and Devices, University of Brighton, Brighton BN2 4GJ, UK; s.sandeman@brighton.ac.uk; 5 ANAMAD Ltd., Sussex Innovation Centre, Science Park Square, Falmer, Brighton BN1 9SB, UK; sergeymikhalovsky@gmail.com; 6 Chuiko Institute of Surface Chemistry, 17, General Naumov St., 03164 Kyiv, Ukraine; 7 Faculty of Natural Sciences, Kazakh National Women’s Teacher Training University, Gogol Str., 114, k. 1, Almaty 050000, Kazakhstan; 8 Laboratorio de MaterialesAvanzados, Departamento de QuímicaInorgánica, Universidad de Alicante, 03690 Alicante, Spain; 9 Laboratory of Engineering Profile, Satbayev University, 122/22, Baitursynov St., Almaty 050012, Kazakhstan; 10 School of Life Sciences, Pharmacy and Chemistry, Kingston University, Penrhyn Road, Kingston upon Thames KT1 2EE, UK

**Keywords:** rice husk, carbon, monoliths, adsorption, PCS, IS, IL-6, IL-8, hemoadsorption, hemocompatibility, biocompatibility

## Abstract

Rice husk (RH) is a renewable siliceous lignocellulosic waste providing a unique, greener and less toxic alternative to conventional synthetic polymeric precursors in the production of carbon-based materials for biomedical applications. In this work we studied the porous structure of RH-lignin-based activated carbon produced in the form of honeycomb carbon monoliths and assessed their potential as hemoadsorbents for blood purification in the treatment of patients with serious medical conditions, such as kidney failure and sepsis. To determine their clinical suitability for such an application, the hemocompatibility and cytotoxicity of the monoliths were investigated using the standard ISO guidelines. The monoliths did not cause any changes in the cell viability or cell lysis. High micro/mesoporosity and surface chemistry of the initial monolith-C, N- and P-doped nanostructured carbon honeycomb monoliths were established by low-temperature nitrogen adsorption (LTNA) studies, mercury porosimetry data (MIP), SEM/EDS analysis and FT-IR spectroscopy. The micro-mesoporous, activated carbon-based filtration/adsorbent prototype devices, in the form of three-dimensional (3D) carbon matrix, functionalized with ion-exchange amino- and phosphate groups and encased in polyolefin heat shrink cable sleeve, have been developed with the capacity to remove protein-bound uremic toxins (PBUTs), such us PCS and IS, as well as inflammatory cytokines (IL-6 and IL-8) from human plasma in a flowing model system. The ammoxidized monolith-N, derived from the monolith-C, had the highest removal efficiency (40.05% for PCS, and 28.4% for IL-6). By contrast, phosphorylated monolith-P demonstrated the highest removal efficiency (54.62% for IS, and 54.4% for IL-8), whilst the monolith-C has the lowest removal efficiency for these adsorbates. These results do not correlate with the LTNA and MIP study results, suggesting that the interaction of surface chemical functional groups with the solutes play key roles in the adsorption mechanism. The ion-exchange mechanism of PBUTs and inflammatory cytokine chemisorption by the monoliths, modified with surface N- and P-containing functional groups, has been proposed.

## 1. Introduction

Chronic Kidney Disease (CKD) has been widely accepted as a major non-communicable human disease affecting over 10% of the adult population in industrialized countries. CKD is mainly caused by metabolic and cardiovascular disorders such as diabetes and hypertension, disproportionally affecting older people, whereas natural kidney aging is driven by age-dependent systemic and renal low-grade inflammation. Moreover, CKD and the associated comorbidities constitute a substantial socioeconomic burden rising with increasing longevity and a growing proportion of older adults in the general population [1].

Major uremic toxins p-cresyl sulfate (PCS) and indoxyl-3-sulfate (IS) are derivatives of p-cresol and indole; those are toxic catabolites, generated by the proteolytic community of intestinal bacteria from the fermentation of amino acids, e.g., phenylalanine and L-tyrosine for p-cresol and tryptophan for indole [2,3]. Specifically, hydroxyphenylacetate decarboxylase catalyzes the formation of p-cresol from phenylalanine and L-tyrosine, whilst tryptophanase catalyzes the formation of indole from tryptophan by the intestinal microbiome, respectively [3]. In the case of CKD, PCS and IS plasma protein conjugates are related to oxidative stress caused by reactive oxygen species (ROS) and cardiovascular disease (CVD) [2,3]. p-Cresol undergoes sulfation by sulfotransferases of colon microflora to form PCS, which can be absorbed through the gut epithelial barrier to enter the bloodstream. Minor amounts of p-cresol and indole can also translocate to the liver from the intestine through a portal vein, where in hepatocyte microsomes, they undergo sulfation by sulfotransferases to form PCS and IS [4]. Specifically, indole is hydroxylated by the cytochrome P450 enzyme to form 3-hydroxyindole (indoxyl) first, which subsequently is sulfated by the SULT1A1 enzyme to form IS [3,5].

As was aforementioned, both PCS and IS have strong affinity for plasma proteins and form conjugates called protein-bound uremic toxins (PBUTs); the latter undergo renal clearance in the renal tubular cells, causing only minor cumulative damage to the kidney [5,6]. Most of these molecules exert biological functions through receptor-mediated signal transduction pathways, including the aryl hydrocarbon receptor (AHR), a sensor, which regulate host immunity [7].

Interleukin-6 (IL-6), a middle molecule toxin with molecular weight of ca. 24 kDa, is the key cytokine mediating age-related inflammation. At the same time, IL-6 has been implicated in the pathophysiology of cardiovascular and renal disorders as a major proinflammatory cytokine [1,8,9]. The accumulation of PBUTs, particularly IS, in cardiomyocytes is linked to increased production of inflammatory cytokines such as IL-6 in cardiorenal syndrome [10]. Interleukin-8 (IL-8) medium middle-molecule, a proinflammatory “chemo-attractant cytokine” (chemokine) with a molecular weight of approx. 8 kDa, plays a central role in chronic inflammatory responses. IL-8 is involved in the pathogenesis of intimal hyperplasia, which is a key mechanism in arteriovenous access stenosis, as well as in atherosclerotic plaque formation. It was also established that the mechanism underlying the upregulation of endothelial IL-8 by IS undergoes through activation of AHR and its association with arteriovenous access complications in hemodialysis (HD) patients [11,12].

Clearance technologies for uremic toxins include hemodialysis (HD) methods and hemoperfusion or hemoadsorption (HA) technology, or a combination strategy of using both HD and HA procedures. During the Low-Flux Hemodialysis (LFHD) process, small-molecule uremic toxins with a molecular weight of ≤500 Da passes through the semi-permeable dialysis membrane with a relatively small average pore size of around 3–5 nm, which restricts the passage of medium-molecule toxins, while High-Flux Hemodialysis (HFHD) uses a dialysis membrane with a larger pore size, usually around 6–8 nm. This allows for a higher flux of solutes, providing HFHD with a better ability to clear medium-molecule toxins than LFHD. However, for larger-sized medium-molecule toxins or those with a very high protein-binding rate (PBUTs) and for some cytokines with a molecular weight close to or above 20 kDa, their clearance efficiency is still not satisfactory. High-cutoff dialysis uses a dialysis membrane with a larger cutoff molecular weight of 50–100 kDa; however, it causes non-selective clearance of albumin, whilst medium-cutoff membrane dialysis uses a membrane with a cutoff molecular weight between that of low-flux and high-cutoff membranes, usually around 10–50 kDa. It can effectively clear small middle-molecule toxins with a molecular weight of 0.5–15 kDa, such as IL-8. Moreover, it also has good clearance effects on some medium middle-molecule toxins with a molecular weight of >15–25 kDa, such as IL-6. HA technology has unique advantages in the clearance of PBUTs due to the use of adsorbents with a specific affinity, i.e., selectively bind to the uremic toxins. In some clinical studies, the combination of HA and HD can achieve better treatment effects for uremic patients; e.g., it was found that the IS and PCS levels in the patients’ blood significantly reduced. HA technology can also be combined with other advanced blood purification technologies, such as high-cutoff dialysis, medium-cutoff membrane dialysis, and hemodiafiltration (HDF). Hence in a randomized controlled study, 136 patients undergoing maintenance HD were divided into four groups: LFHD, HFHD, HDF, and HA + LFHD. The results showed that, for toxins with a medium protein-binding affinity (e.g., IS and PCS), HDF (41–47%) and HA + LFHD (45–49%) demonstrated superior removal capabilities to HFHD and LFHD [8]. Overall, the study concluded that combining HD with biweekly HA therapy can significantly reduce inflammatory markers and improve symptoms in end-stage renal disease (ESRD) patients.

However, high-cutoff dialysis also has some problems. One of the main issues is the non-selective clearance of albumin [13].

The adsorptive therapy represents an innovative strategy for addressing uremic toxin removal in CKD patients; it relies on the ability of certain adsorbent materials to selectively eliminate PBUTs from the bloodstream, not only in the free fraction, but also the protein-bound fraction, due to their high affinity for these molecules. The mechanism of action involves the interaction between PBUTs and adsorbent materials. When the patient’s blood or dialysate flows through an adsorptive therapy device, uremic toxins bind to the adsorbent surface due to chemical and physical forces. Once bound, they do not detach, causing material saturation depending on the surface [10]. The adsorption of proteins is also a common phenomenon in the presence of nanomaterials in biological environments and depends on the size, shape, chemical composition of the sorbent surface, and protein structure. Positive-negative charge attraction between a cytokine and nanomaterials’ surface may be one of the main events, ruling the adsorption mechanism. The interaction between chemokines and the chemically modified surface of nanomaterials defines the potential to remove excess of chemokines through selective adsorption; in fact, various nanomaterials surfaces may have an affinity for IL-8, now called Cys-amino acid chemokine X-Cys ligand (CXCL8). In a research paper regarding methods for extracorporeal detoxification from proinflammatory mediators, it was demonstrated that graphene nanoplatelets are valid adsorbents to remove several proinflammatory cytokines, including IL-8 [11,14].

Activated carbon (AC), made from a wide range of natural and synthetic precursors, was among the earliest materials used for extracorporeal blood purification and remains a fundamental component in current treatment methods. Among the adsorbent materials used is nanoporous carbon (NPC), which significantly improves toxin clearance when used simultaneously with conventional HD or with hemoperfusion (HP). NPC has a high specific surface area and exceptional adsorptive properties. NPC’s high adsorption capacity and other adsorbent materials have led to a significant reduction in toxin concentrations in CKD patients [10]. Regarding the available sorbent materials, NPC has been one of the most studied sorbent materials in the past because it has a high specific surface area and exceptional adsorptive properties, which leads to significant reduction in toxin concentrations in CKD patients and significantly improves toxin clearance when used simultaneously with conventional HD. Hence, a carbon-silica-based NPC adsorbent CMK-3 was developed by nanocasting methods and exhibited two distinct pore domains, i.e., micropores and mesopores. Moreover, it demonstrated high adsorption capacity towards small water-soluble toxins (creatinine), protein-bound molecules (IS), middle molecules (β-2-microglobulin) and cytokines of different size (IL-6 and IL-8) [9,15].

NPC adsorbents offer considerable advantages over the polymer-based systems. They outperform other adsorbents through the existence of a highly developed internal surface area which is suitable for non-specific adsorption of predominantly hydrophobic organic molecules. Early studies using various granular NPC formulations from a range of natural sources showed some success, but were ultimately constrained by variable performance, poor hemocompatibility particularly related to platelet loss, pressure drop, and poor adsorption of larger biological molecules when coated. This structure can be maintained, and the potential for pressure drop can be reduced by the production of NPC monoliths, formed *as extruded* cylinders with numerous micro-diameter channels along which blood flows [16]. However, a monolith synthesis route which enabled the production of monoliths with attrition resistance and adsorptive capacity on scale up to a clinically relevant size was not possible using the previous synthesis route [17].

As a natural siliceous lignocellulosic precursor, rice husk (RH) is an abundant agricultural by-product with minimal commercial value. It is traditionally burned or disposed of in landfills in rice-growing countries; however, RH incineration leads to air pollution and is ineffective due to its low calorific value and high ash silica content which makes RH poorly biodegradable [18]. Converting RH into NPCs with good adsorption properties not only could add substantial value to the resulting composites, such as carbon/silica adsorbents and catalysts [19,20,21], but also reduce the inherent problems associated with the current methods of this biomass waste disposal, offering a “greener technology” approach. Recently, there has been a renewed interest in utilizing RH-based NPCs as a low-cost sorbent for removing organic pollutants and heavy metals from aqueous media [18,22]. In terms of potential advantages, it is a renewable bioavailable material with a low content of potentially toxic substances, which is important for biomedical applications. The nanoscale silica phytoliths contained in RH serve as a template to create additional meso/macropore space in NPCs within the nanoscale range [23,24,25].

Porous adsorbents—ranging in pore size from nanometers to micrometers—are often fabricated from both natural and synthetic sources and highly purified NPC can be made hemocompatible. The key performance metrics of any adsorbent include adsorption capacity, selectivity, kinetics, biocompatibility and cost-efficiency. In hemoperfusion, blood is drawn from the patient and passed through a column containing activated carbon or other microporous materials. This selective adsorption process is crucial for effectively removing harmful substances from the blood without damaging the formed elements of blood [26].

In this study we explored the possible use of NPC honeycomb monoliths, obtained from RH and phosphorylated RH powders and lignin, as a hemoadsorbent for blood filtration applications to treat patients with a range of serious medical conditions, such as kidney failure and sepsis. Previously we showed that the carbonized RH composites in the form of NPC powder or monoliths adsorb a variety of medium- and large-weight biological toxins, such as bacterial lipopolysaccharides (LPSs) [18], otherwise called endotoxins, which are components of the cell wall of Gram-negative bacteria and are a key pathogenic factor in inducing sepsis. LPS can activate Kupffer cells in the liver and induce the production of IL-6, IL-8, and other inflammatory cytokines [27].

Medical devices such as HP columns that are used in direct blood contact require evaluation of their hemocompatibility before clinical use. The suitability of the monoliths for use in a hemoperfusion medical device in contact with patient blood was evaluated following the international standard tests for in vitro cytotoxicity and hemocompatibility, ISO 10993-4/5 [28,29]. Hemocompatibility was assessed by a selection of tests including platelet activation, coagulation, complement activation, granulocyte/monocyte activation and hemolysis following circulation of healthy donor blood though the monolith. Following the recommendations in the literature, the test methods and conditions used were optimized to provide stable and reproducible results for the intended use of monoliths in a dynamic system [30].

The aim of the current study is also to design novel 3-dimensional multifunctional devices for hemoadsorption in the form of encased-in-polyolefin-heat-shrink-cable-sleeve nanostructured carbon honeycomb monoliths produced from RH and lignin, and assess their hemocompatibility and cytotoxicity using ex vivo healthy donor HP, as well as their effectiveness in eliminating PBUTs (PCS and IS) and inflammatory cytokines (IL-6 and IL-8) from human plasma in a flowing recirculation system.

## 2. Results and Discussion

### 2.1. Honeycomb Carbon Monolith Geometry Design, Metric Parameters and Function

**Figure 1:**
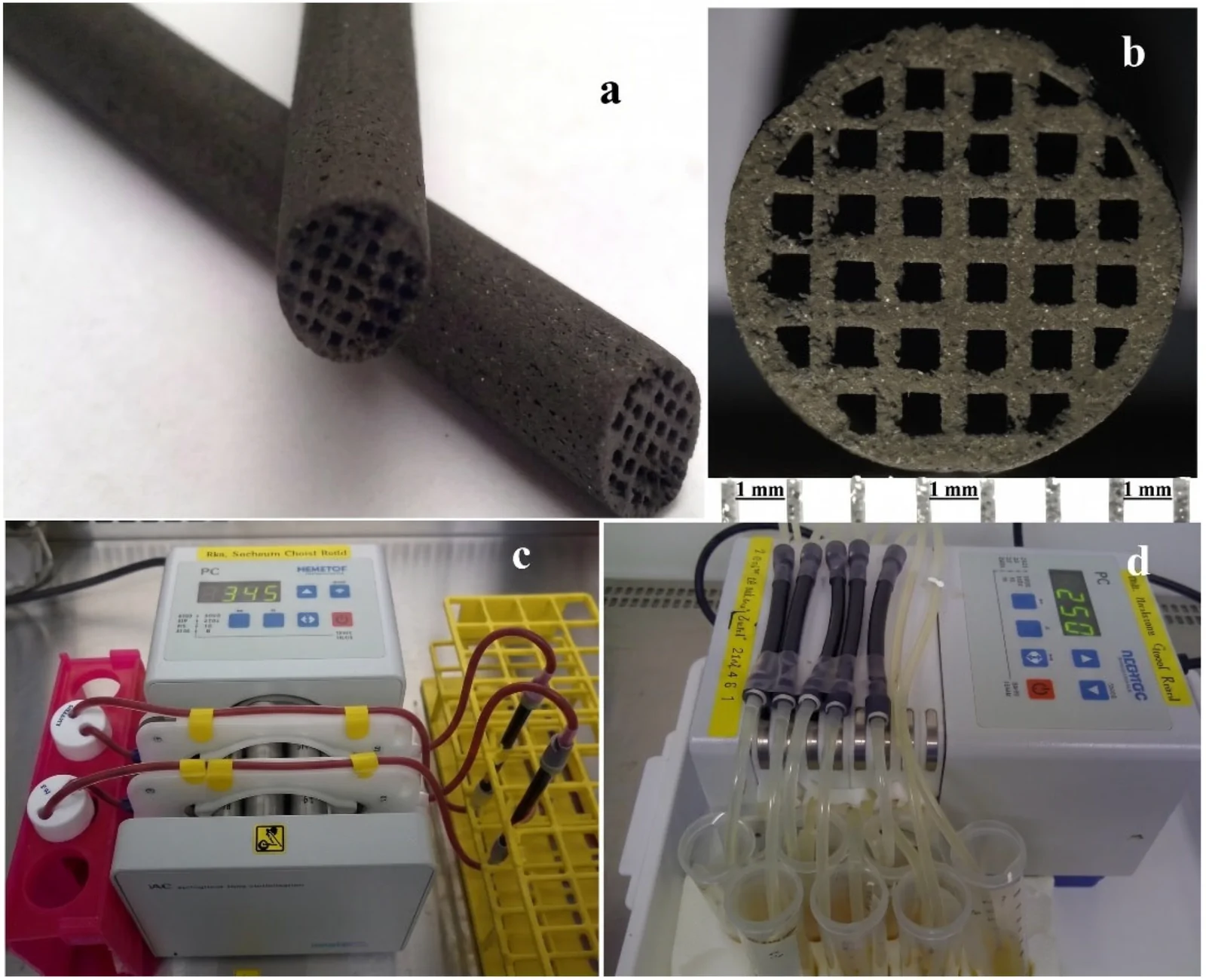
Honeycomb carbon monoliths: (**a**) external appearance of as-prepared CDA monoliths—10 cm length; (**b**) geometry of cross-section area: number of channels—32; cell diameter—0.6 mm; wall diameter—0.4 mm; (**c**) experimental system for continuous blood circulation through monoliths for hemocompatibility testing; (**d**) experimental system for continuous plasma circulation through monoliths for evaluating toxin removal efficiency.

The geometry and design of extruded “green” monoliths is defined by the type of a dye plate; in our experiments, initial “green” monoliths had a diameter of 10 mm, but their linear parameters decreased more than 30% due to shrinkage in the course of carbonization. The small as-prepared cylindrical prototype monolith-C with the channel structure through which blood would flow within the HP device is shown in Figure 1a–d.

The transverse section through the CDA monolith in Figure 1b shows that it contains 32 cells (24 square- and eight triangular-shaped) within the circular area of 6.8 mm in diameter, with cell diameters of 0.6 mm and wall diameters of 0.4 mm. The shrink-wrapped 10 cm in length monolith was connected with standard sterile syringe tips to a peristaltic pump, and the blood was recirculated through the channels (Figure 1c). Assuming that the area of a triangular-shaped cell for eight peripheral channels is approximately half as large as the area of a square cell, the total volume of channels (V) within a monolith can be estimated according to Equation (1):

V ≈ (m + 0.5n) × L× d^2^ (1)

where m is the number of square cells (24); n is the number of triangular-shaped cells (8); L is the length of a monolith (10 cm); and d is the cell diameter (0.6 mm; 0.06 cm). According to Equation (1), the volume of channels is approximately 1.008 cm^3^ ≈ 1 mL [18].

### 2.2. Physicochemical Investigation of Rice Husk-Derived Honeycomb Carbon Monoliths

#### 2.2.1. Low-Temperature Nitrogen Adsorption and Mercury Intrusion Porosimetry

**Figure 2:**
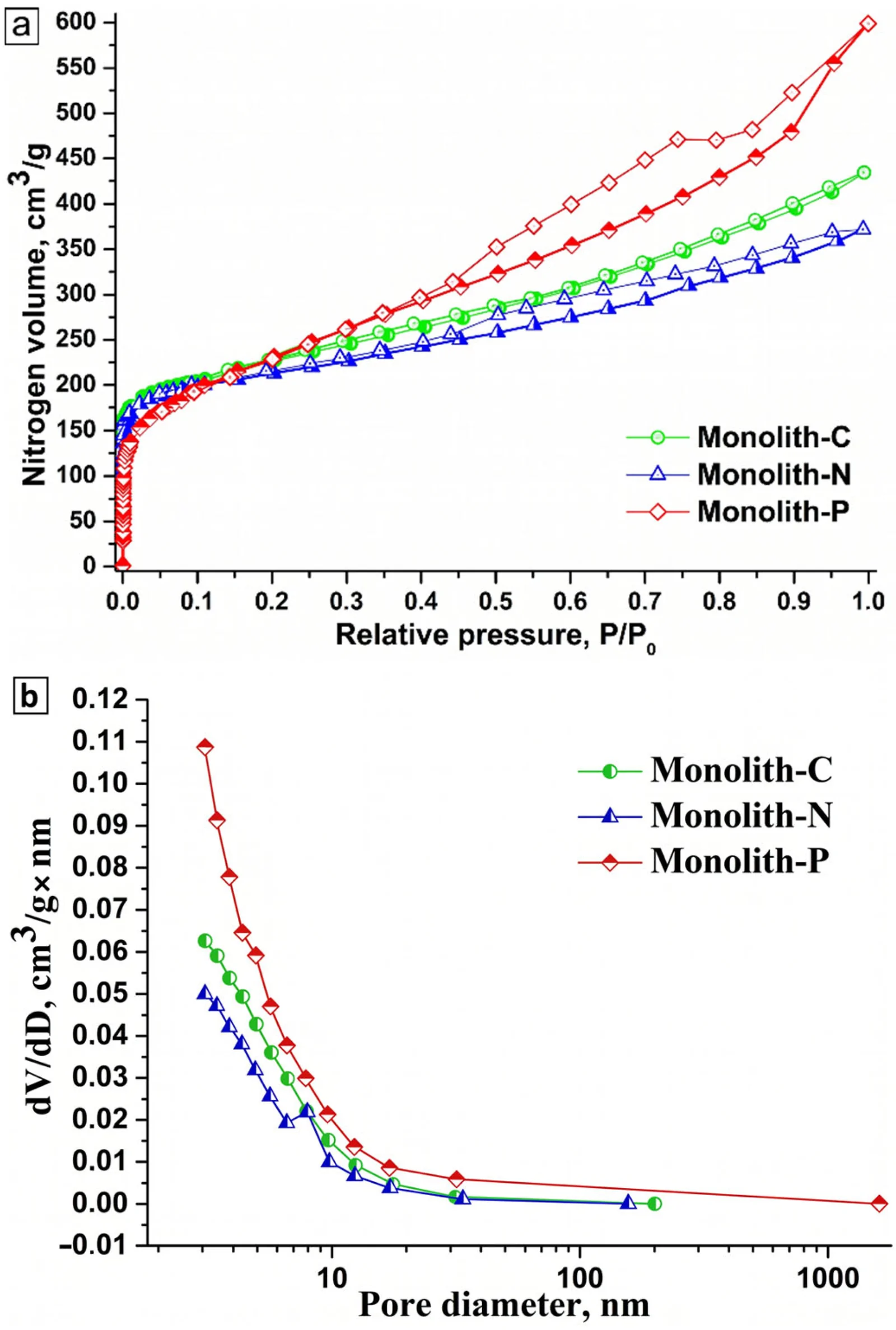
BJH–pore size distribution curves (**a**) derived from nitrogen adsorption–desorption isotherms; (**b**) data for the nanostructured carbon honeycomb monoliths.

**Figure 3:**
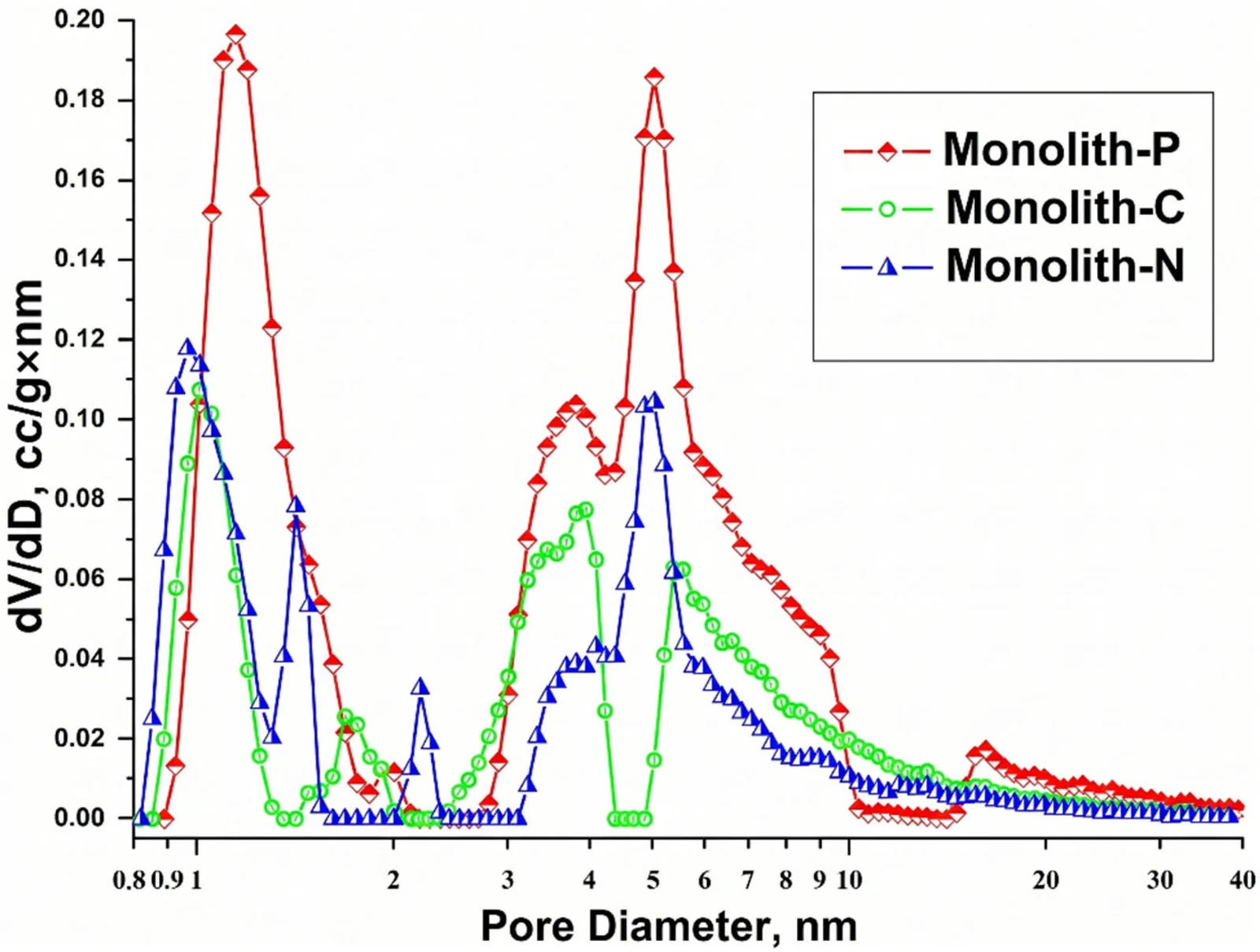
QSDFT pore size distribution curves for three monoliths.

The nitrogen adsorption–desorption isotherms (Figure 2a) and pore size distribution (PSD) curves, calculated from nitrogen adsorption–desorption isotherms using the Barrett–Joyner–Halenda (BJH) method (Figure 2b) and the slit-cylindrical quenched solid density functional theory (QSDFT) equilibrium model (Figure 3), are summarized in Figure 2 and Figure 3, respectively.

According to the IUPAC classification [31], the N_2_ adsorption–desorption isotherms of all three monoliths are a mix of type I (at the beginning of the isotherm) and type IV (at the end of the isotherm). However, a notable steep slope at low relative pressure (P/P_0_ ≤ 0.1) presented in the type I (a) isotherm for the monolith-C and monolith-N samples indicates abundant narrow micropores (width < 1 nm), while monolith-P sample, presented in the I (b) type isotherm, were associated with a broader range of pore size distributions (Figure 2a), including wider micropores and possibly narrow mesopores (<2.5 nm). Each isotherm, a mix of H3 and H4 types, also shows a distinct hysteresis loop at intermediate-to-high relative pressures, which is characteristic of the presence of slit-shaped large micro- and small mesopores.

From Figure 2b it is also evident that capillary condensation occurs due to the presence of mesopores in the monoliths, according to the BJH analysis method, which is confirmed by the shape of the hysteresis loop formed by the adsorption and desorption branches of the isotherms (Figure 2a). As shown in Figure 3, the QSDFT PSD curves reveal that the porous structure of the studied monoliths is characterized by polymodal distribution with peaks in both micro- and mesopore size ranges. The PSD curves within supermicropore range for monolith-C and monolith-N have bimodal character with peaks at 1.2, 1.7 nm and 0.9, 1.4 nm, respectively; whereas monolith-P has a dominant broad peak at 1.2 nm. All three monoliths have pronounced multimodal mesopore size distribution within the 2–10 nm range, with peaks at 3.7 and ~5 nm, while the monolith-N sample has a pronounced peak at about 2.2 nm. The PSD curves obtained by the QSDFT method revealed hierarchical micro/mesoporous structures in all monolith samples (Figure 3).

The surface characteristics of the monolith samples obtained from low-temperature nitrogen adsorption studies are summarized in Table 1.

The textural characteristics of the obtained PC samples are shown in Table 1: S_BET_ is the Brunauer–Emmett–Teller (BET) theory specific surface area; S_QSDFT_, V_QSDFT_ and D_QSDFT_ are the specific surface area, the total pore volume, and the average pore diameter, calculated by the quenched solid density functional theory (QSDFT) method, respectively; and V_BJH_ is the mesopore volume, calculated using the Barrett–Joyner–Halenda (BJH) method. V_DR_ and D_DR_ are the micropores volume and diameter, calculated using the Dubinin–Radushkevich (DR) method. V_MIPs_ are pore volumes determined by mercury intrusion porosimetry analysis (MIP).

In Table 1 it is shown that there is a correlation between the textural characteristics of the initial and modified adsorbent materials; e.g., the monolith-N was derived from the monolith-C via oxidative ammonolysis, while the monolith-P was produced from the CRH-P-450 powder and mixed with lignin via extrusion, followed by carbonization. The S_DFT_ values are slightly higher, but on average, they correspond to the S_BET_ values. Due to the functionalization of the Monolith-C surface, a slight decrease in the textural parameters is observed. Surface oxidation followed by amination resulted in only a minor decrease in the specific surface area (816 to 790 m^2^/g) and micropore volume (0.407 to 0.381 cm^3^/g), indicating preservation of the carbon framework. However, a noticeable reduction in mesopore volume was observed, with the BJH pore volume decreasing from 0.382 to 0.292 cm^3^/g. Hence, the specific surface area of the Monolith-N decreases by 5–10% on average, compared to the original; the total pore volume calculated by the DFT method decreases from 0.617 cm^3^/g to 0.542 cm^3^/g (by 12%), while the mesopore volume calculated by the BJH method decreases from 0.334 cm^3^/g to 0.286 cm^3^/g (by 14%). This effect was even more pronounced for pores below 50–100 nm according to mercury intrusion porosimetry. These results suggest that nitrogen functionalization mainly affected the accessibility of mesoporous channels through partial pore narrowing or blockage rather than structural collapse of the monolithic carbon network.

A similar picture is observed in the case of mercury porosimetry (MIP) data—the volumes of meso- and macropores decrease. Monolith-P contains a higher number of mesopores, while Monolith-C has a higher number of micropores. Overall, Monolith-P has superior pore volume and surface area to Monolith-C (see Section 3. Materials and Methods).

**Figure 4:**
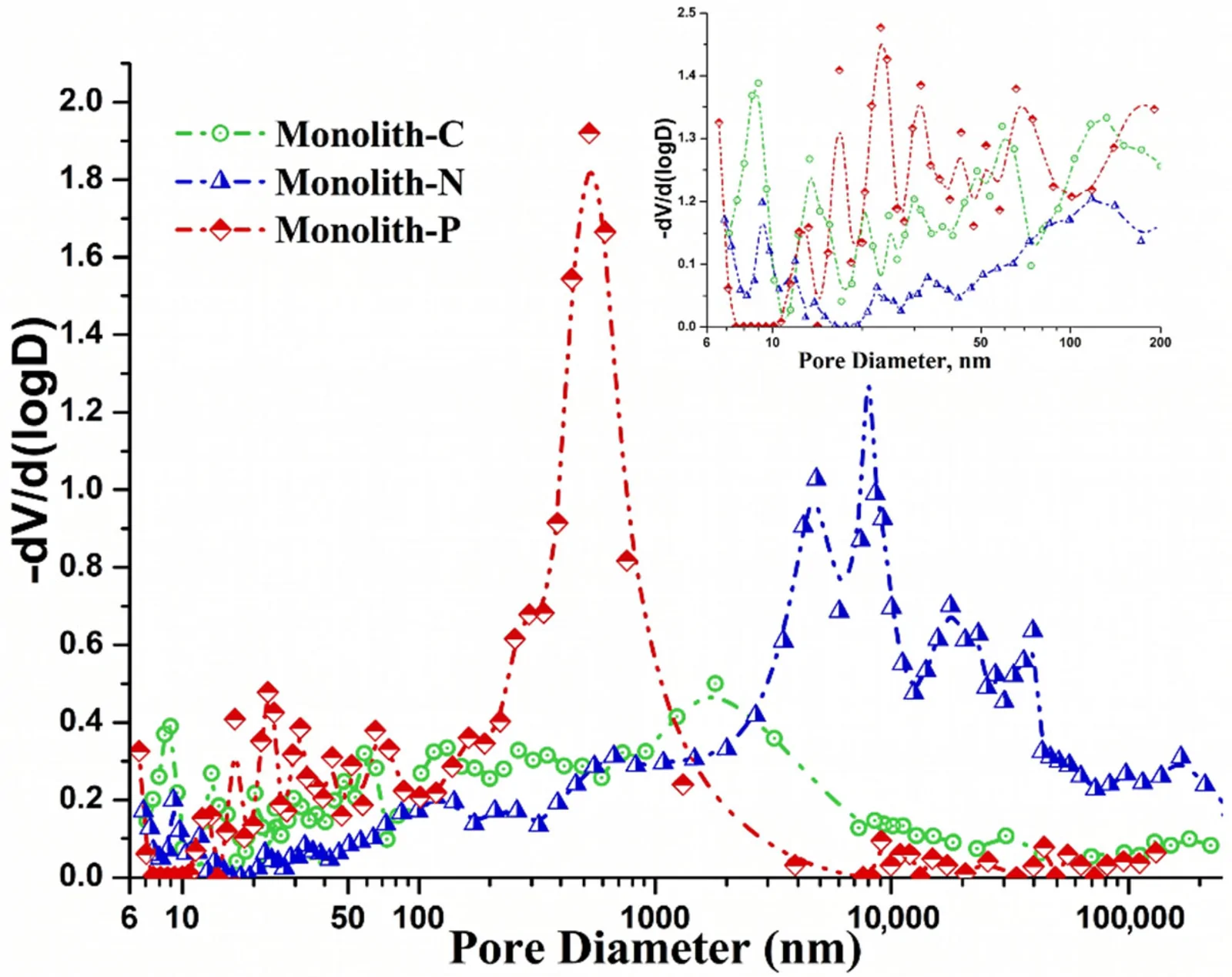
Pore size distributions in carbon monoliths according to the mercury intrusion porosimetry.

MIP PSD curves in carbon monoliths are presented in Figure 4; the graphs indicate the presence of pores with varying sizes in all the samples. Monolith-C has a pronounced peak in the range of approximately 1–2 µm, with an additional contribution of macropores size within the range of 50–1000 nm. A multimodal PSD, dominated by macropores in the range of 4–50 µm was observed for Monolith-N, specifically the main maximum was located near 8–10 µm, accompanied by several secondary peaks. A narrow PSD with a pronounced maximum at approximately 600 nm was observed for Monolith-P. The contribution of pores larger than 2 µm was negligible, indicating a relatively homogeneous porous network.

#### 2.2.2. Surface Structure and Morphology and the Elemental Content of the PC Components According to SEM/EDS and CHNOS Analyses

**Figure 5:**
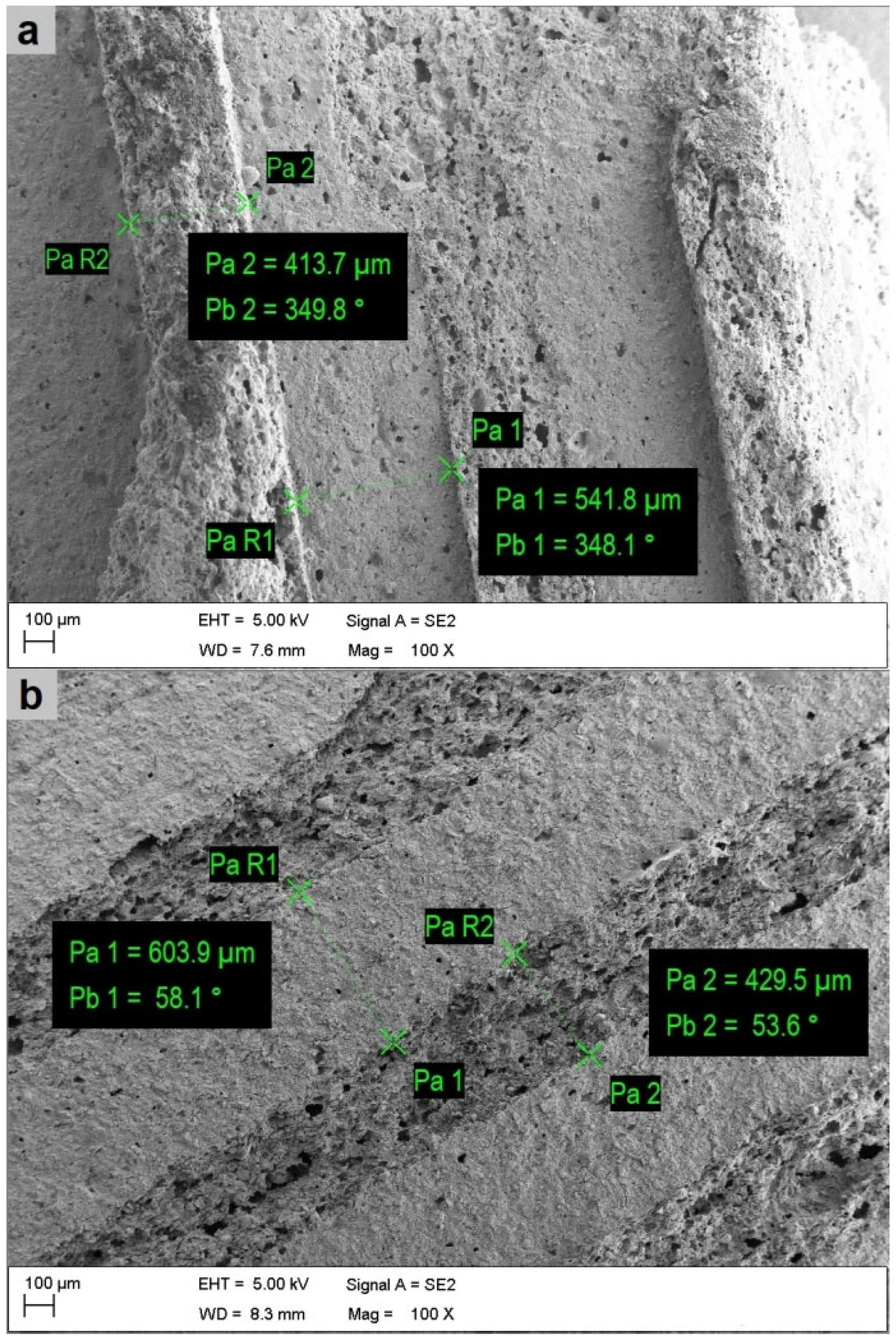
SEM images of an extruded monolith longitudinal section: (**a**) 413.7 µm and internal channel with the size of 541.8 µm; (**b**) 429.5 µm and internal channel with the size of 603.9 µm.

**Figure 6:**
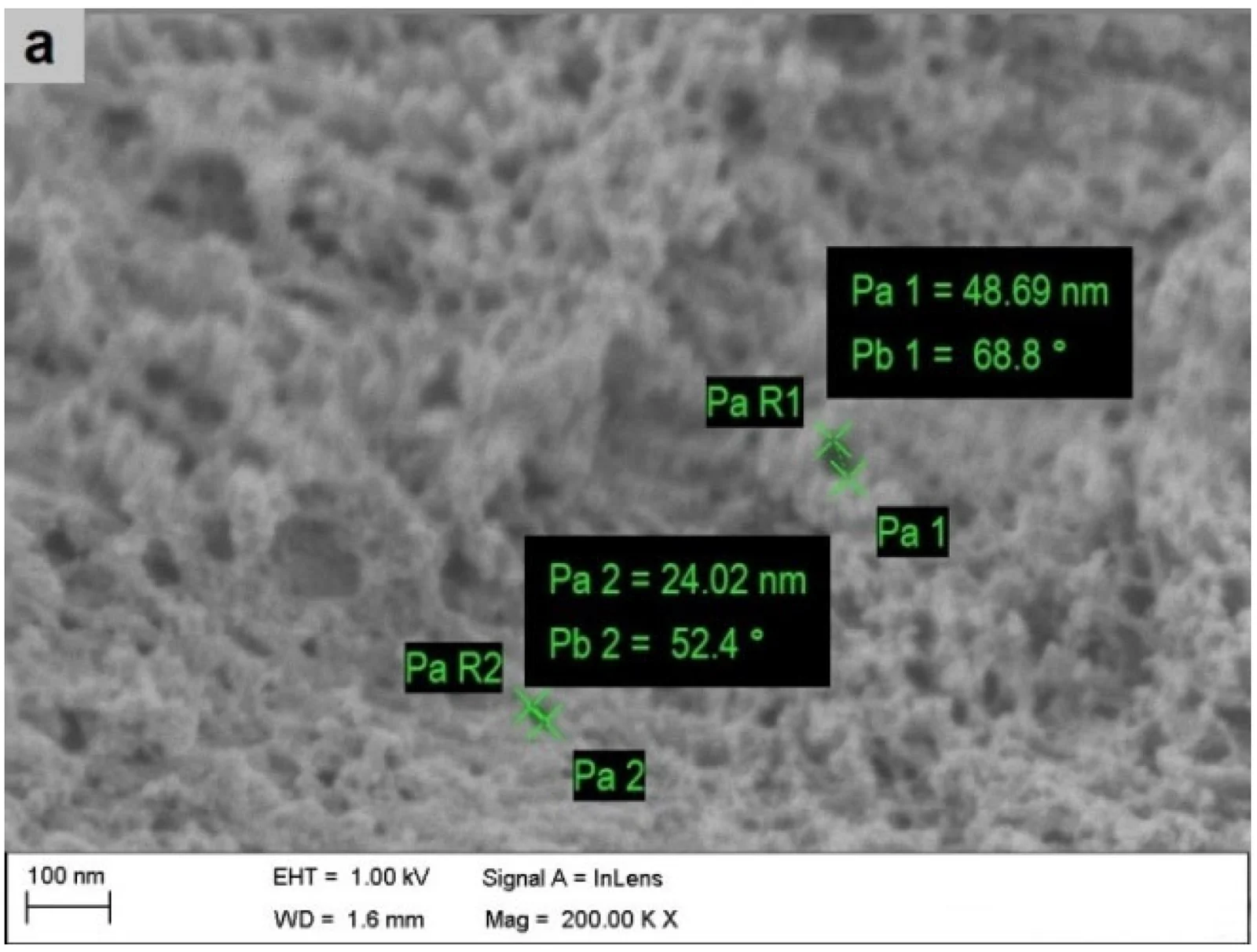

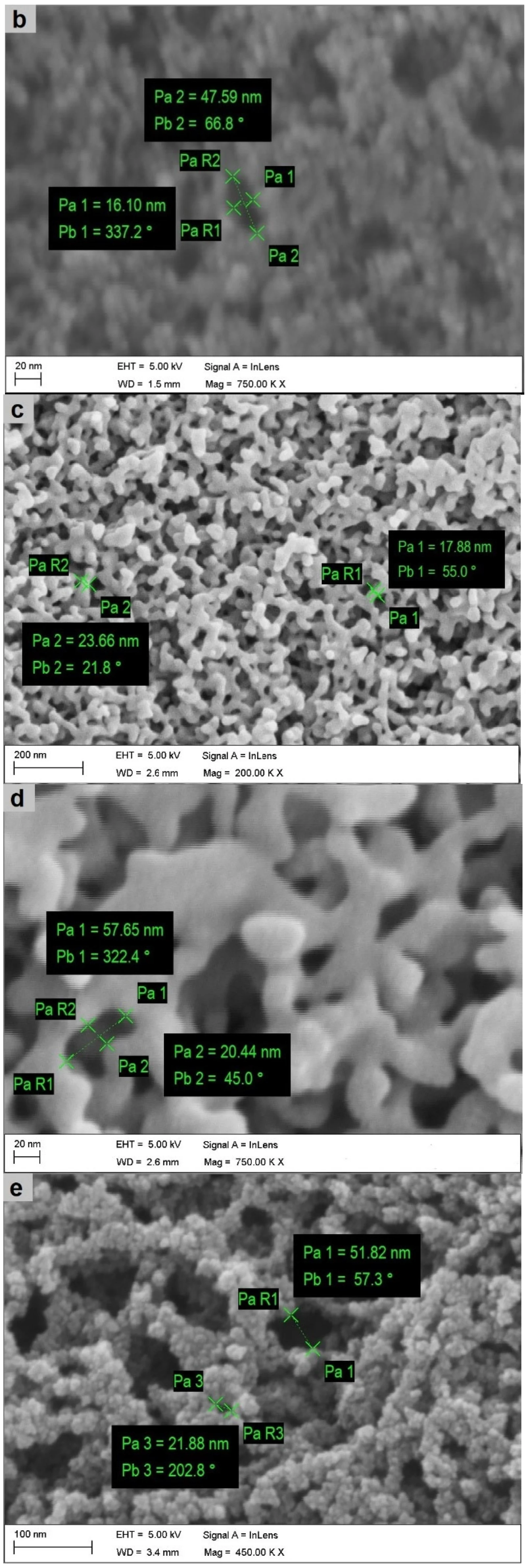

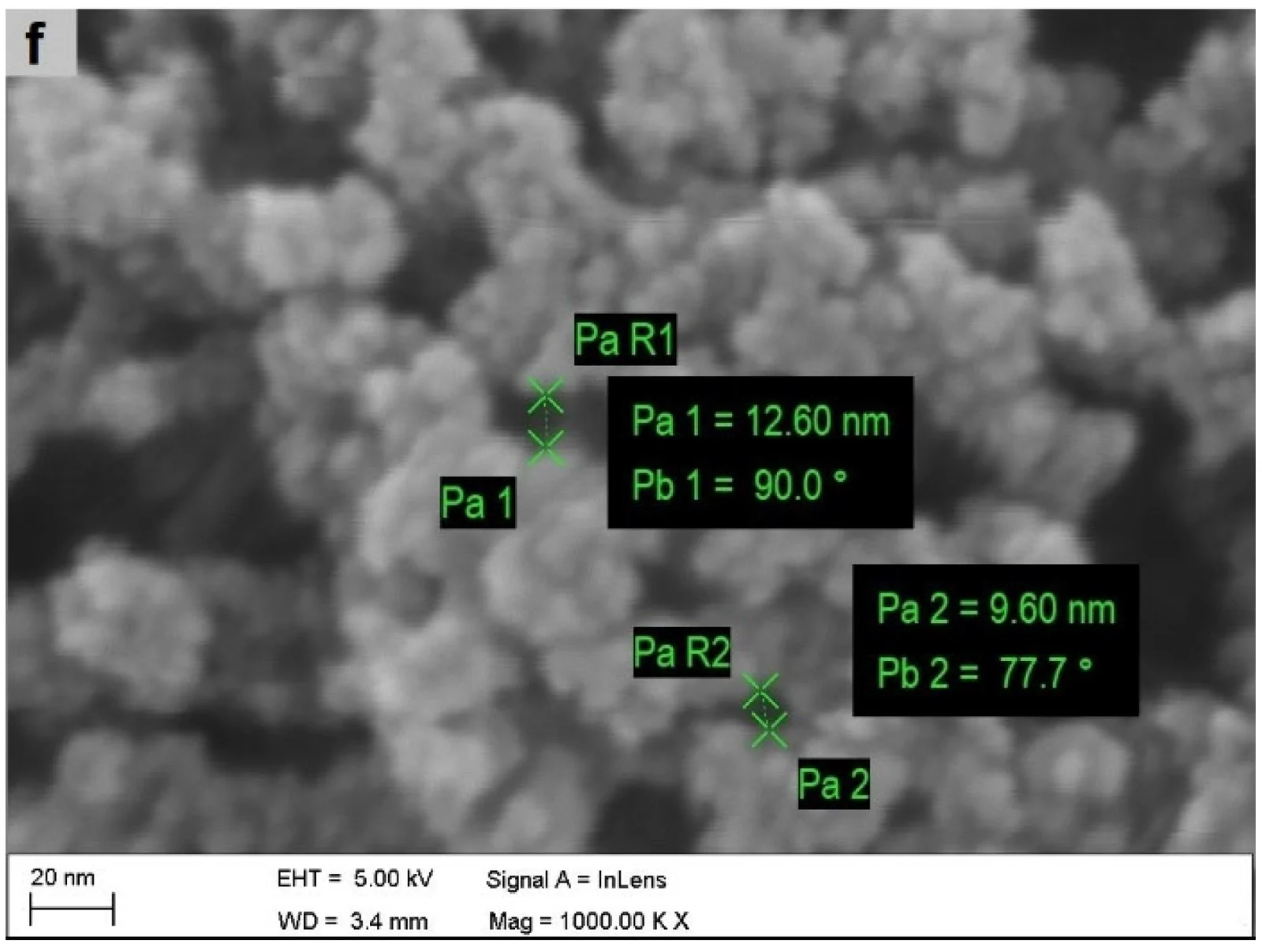
SEM images of: monolith-C/monolith-N internal walls (**a**,**b**); monolith-C/monolith-N intact channels (**c**,**d**); monolith-P (**e**,**f**) at different resolution.

The SEM images of the resulting monoliths, shown in Figure 5, demonstrate the porous nature of the carbon monoliths. Low-magnification images in Figure 5a,b demonstrate the basic morphology of the longitudinal section of monoliths, with visible structural differences in the form of extruded (walls) and internal (channel) structures. Higher-magnification images in Figure 6 demonstrate the basic morphology of the porous internal structure in the range of small macropore and mesopore diameters, respectively. Images of the surface at sites of monolith wall fracture (Figure 6a,b,e) and the internal channel surface were taken (Figure 6c,d,f).

The SEM image of monolith-N (Figure 6b,d) shows channels in the porous-material transport macropores. Despite the oxidative ammonolysis of monolith-C (Figure 6a,c), monolith-N retained the spongy macrostructure typical of RH-derived carbon nanomaterials. Interconnected open meso- and microporous structures can promote mass transport, leading to improved adsorption capacity [32]. The presence of mesopores and three-dimensional interconnected porous structures can reduce diffusion resistance. SEM images show that monolith-P (e, f) retained the characteristic morphological structure typical of carbon nanomaterials derived from RH despite harsh chemical treatment. The spongy surface of the monolith-P is characterized by a well-developed mesoporous structure.

The monoliths-N, treated with HCl (monolith-N_HCl) and washed with distilled water to remove chemically unbound nitrogen (ammonia), were examined using energy-dispersive X-ray spectroscopy (EDS) microanalysis using a scanning electron microscope. According to EDS semi-quantitative microanalysis of the nitrogen content after acid treatment and washing, the nitrogen content decreased from 3.7 to 3.3%, which indicates the chemical stability of nitrogen-containing groups formed on the surface, as shown in Figure S1a,b (see Supplementary Materials Section).

According to the results of EDS-microanalysis, initial precursor CRH-P-450 powder (Figure S1c) consists mainly of carbon (92.4%), oxygen (3.5%), and phosphorus (2.5%), and contains only trace amounts of residual silicon (0.1%), sulfur (0.1%) and unwashed sodium (1.3%) impurities. While the resulting monolith-P (Figure S1d), produced from the CRH-P-450 and lignin binder precursors, which is rich in carbon, consists mainly of carbon (96.6%), oxygen (1.7%), and phosphorus (1.4%) and contains only trace amounts of residual silicon (0.2%) and sulfur (0.1%) impurities. The increase of carbon content and decrease of phosphorus content in the resulting monolith-P, produced from the CRH-P-450, can be explained by the use of lignin binder precursors, which is rich in carbon (compare Figure S1c,d).

For a more accurate assessment of nitrogen content in the monolith-N, CHNOS analysis is used. According to the results of CHNOS elemental analysis, initial precursor monolith-C (Table S1, Figure S2a) consists mainly of carbon (92.15%), nitrogen (0.02%), hydrogen (1.89%) and oxygen (5.71%). While the monolith-N, after oxidative ammonolysis of monolith-C consists mainly of carbon (86.7%), nitrogen (3.03%), hydrogen (1.88%) and oxygen (8.12%). After treatment with concentrated aqueous HCl, the resulting monolith-N_HCl consists mainly of carbon (79.36%), nitrogen (2.93%), hydrogen (1.44%) and oxygen (16.05%) as shown in Table S1 and Figure S2c. The data presented in Table S1 also validate that during the oxidative ammonolysis of monolith-C, the nitrogen content increased to 3.03%, while after acid treatment, it decreased to 2.93%. This indirectly indicates the predominant formation of strong covalent bonds between nitrogen and carbon. Specifically, the nitrogen is present primarily in the form of nitrogen-containing groups, such as NH_2_ amino groups on the carbon surface, since imine and amide groups are easily hydrolyzed by hydrochloric acid. This likely accounts for the slight decrease in nitrogen content (0.1%) in the monolith-N_HCl sample, compared to the monolith-N sample.

#### 2.2.3. FT-IR Spectroscopy of Surface-Functional Nanostructured Carbons

**Figure 7:**
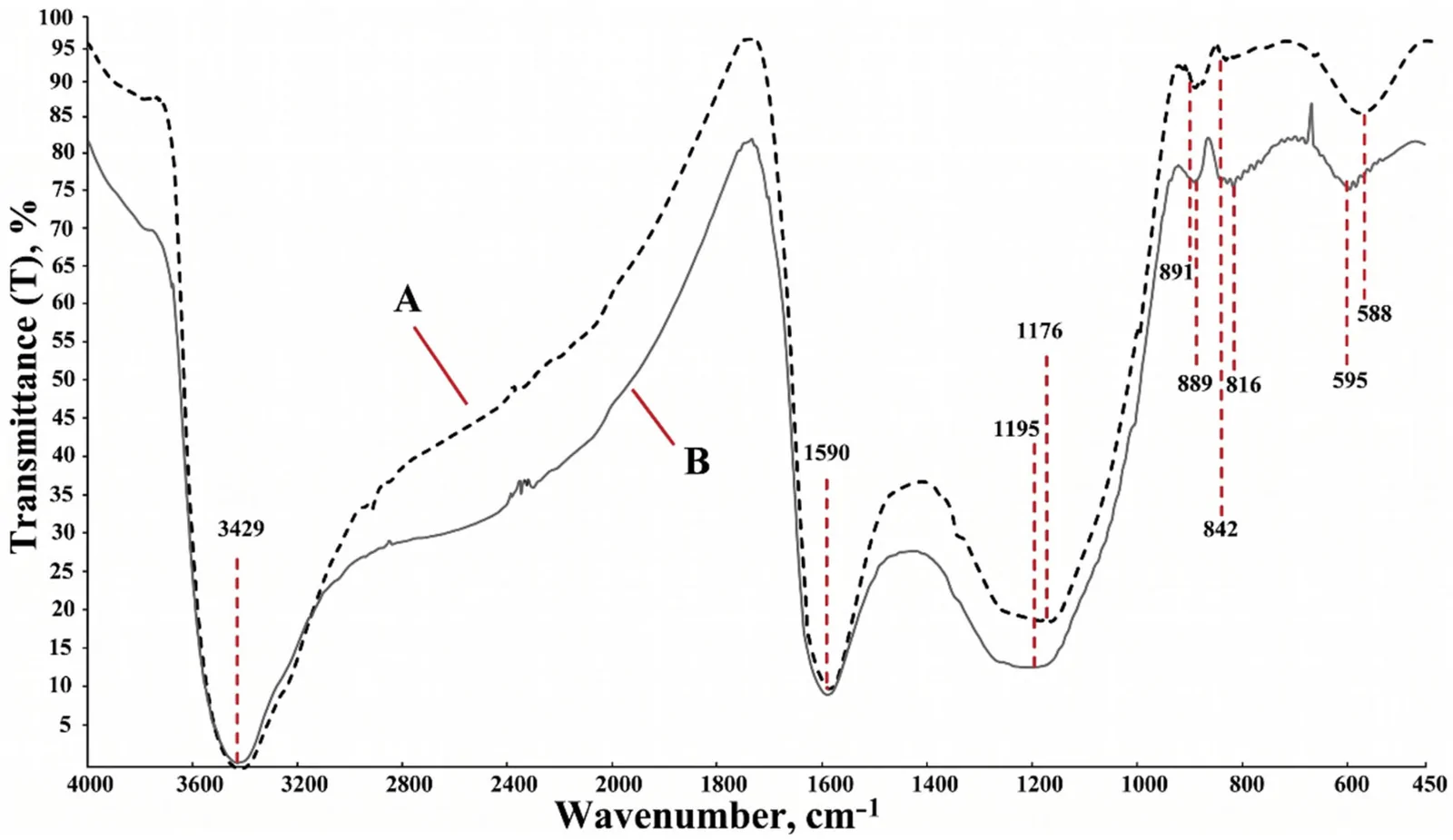
FT-IR spectra—monolith-N before and after acid treatment: A—monolith-N; B—monolith-N_HCl.

Figure 7 shows the FT-IR transmission spectra of the monoliths after the oxidative ammonolysis reaction (Figure 7, line A) and after treatment with HCl solution (Figure 7, line B).

It has been previously reported that N-doped porous carbons, like the monolith, show typical peaks at 3600–3100 cm^−1^ that correspond to the O–H stretching vibrations.

The presented spectra have a complex shape and consist of several overlapping groups, indicating that the sample may contain nitrogen in several forms. The FT-IR spectra of the samples obtained exhibit an increase in the frequency of peaks characteristic of vibrations of nitrogen-containing groups: the N-H (deformation), C=N (stretching), and skeletal vibrations of aromatic rings—region: 1560–1590 cm^−1^, and the C-N (stretching) and νC–O of C–OH ~1190 cm^−1^, attributed to alcohol and/or carboxyl functional groups, are characteristic of all carbon adsorbents [33].

**Figure 8:**
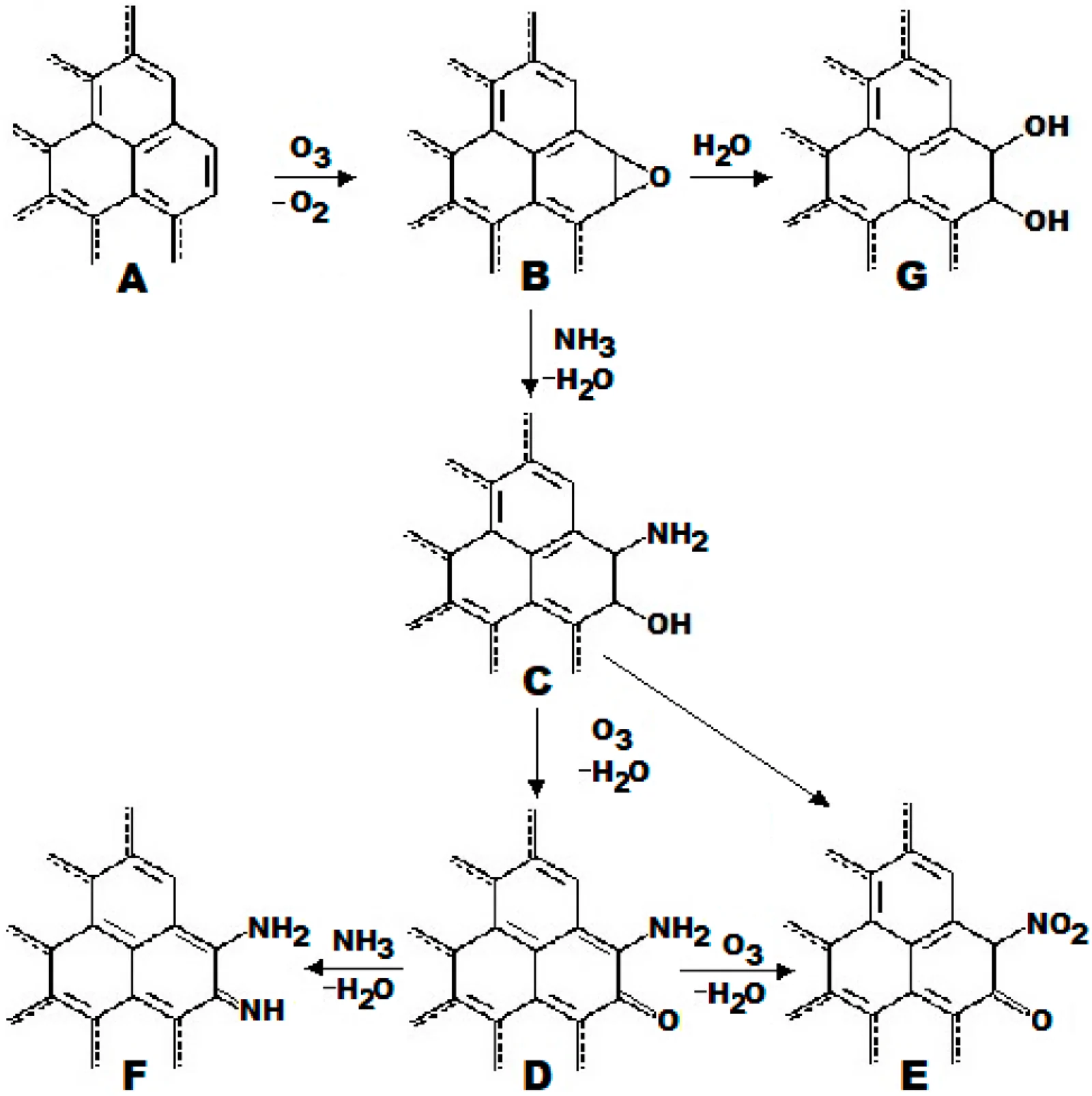
A plausible mechanism of reaction scheme for the incorporation of nitrogen into a carbon matrix during low-temperature oxidative ammonolysis [32].

After acid treatment, no significant changes are observed in the spectrum of the monolith-N, compared to the spectrum of the monolith-N sample, which was treated with HCl, indicating the stability of the grafted nitrogen-containing groups on the carbon surface of the monolith-N, and is consistent with the pattern of changes in the nitrogen content, according to elemental CHNOS analysis data, as described in Section 2.2.2 (see the Table S1). It also means that the monolith-N sample is expected to have a considerable amount of oxygen-containing functional groups (Table S1), as interpreted by a proposed reaction mechanism of the monolith-C surface ammoxidation, according to a scheme depicted in Figure 8.

From the proposed mechanism of the monolith-C surface chemical transformations, to form functional groups in the course of ammoxidation reactions to yield the monolith-N, demonstrated on the scheme in Figure 8, it follows: (**A**) fragment structure of graphene edge of the monolith-N precursor (the monolith-C, as described in Section 3); (**B**) fragment structure of epoxide (oxirane) functional group, formed due to ozonation of fragment structure **A**; (**C**) fragment structure of amino phenolic (hydroxylic and amino-) functional groups formed upon nucleophilic attack of fragment structure **B** with ammonia in the course of ammoxidation; (**D**) fragment structure of keto group, derived from oxidized phenolic group (fragment structure **C**) by an excess of ozone adsorbed on the carbon surface; (**E**) fragment structure of surface nitro group, formed by the ultimate oxidation of amino group in fragment structure D with ozone adsorbed by the carbon surface; (**F**) fragment structure of aminated carbonyl groups in fragment structure D with excess of gaseous NH_3_; and (**G**) “catechol-like” fragment structure, created by hydrolysis of epoxide (oxirane) functional group in fragment structure B.

**Figure 9:**
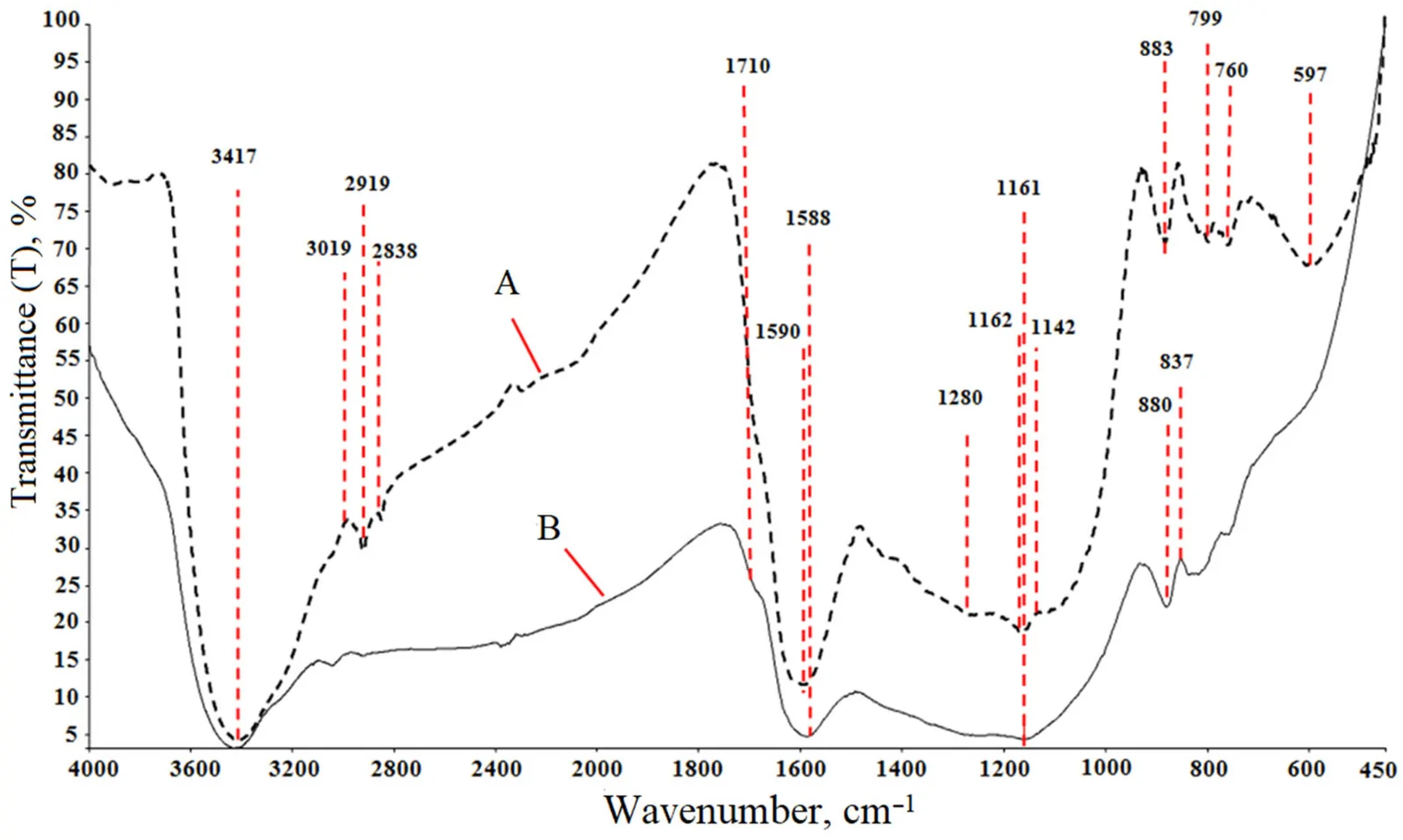
FT-IR spectra of the samples CRH-P-450 (A) and the monolith–P (B).

FT-IR spectra of CRH-P-450 and Monolith–P material samples, shown in Figure 9, differ from each other in the number of absorption bands, their location, and intensity, which indicates a change in the chemical composition and functional coating of the surface of the obtained carbon materials during the modification process.

The FT-IR spectra of both samples contain a broad, intense signal of OH-group stretching vibrations in the region of 3600–3200 cm^−1^, intense absorption bands of C=C groups of aromatic rings at 1580–1620 cm^−1^ and C=O carbonyl groups with a maximum at 1707 cm^−1^, as well as absorption bands at 1710 cm^−1^ and 1680 cm^−1^ of varying intensity, indicating the possible presence of C=O groups of aromatic acids, aldehydes, ketones, diketones, lactones, ketoesters, etc., in the materials. The low-intensity absorption band at 1710 cm^−1^ is explained by the possible presence of C=O (carbonyl) in the structure of the material as part of ketone or aldehyde groups on the surface of the sorbent. In the spectrum region of 1270–1000 cm^−1^ there are several absorption bands of medium intensity, which do not exclude the presence of C–O bonds of esters, alcohol, carboxyl, phenolic and alkoxyl groups. There are also bands in the range of 1050–1120 cm^−1^, with a maximum at 1140 cm^−1^ related to the stretching vibrations of CO in the composition of carboxyl, as well as conjugated C-O groups: C=C–C-O; O=P–O-C–, etc. The spectra contain bands that characterize the presence of phosphate groups, 1280 cm^−1^ –P=O and 1140 cm^−1^ –P-O-C, which is consistent with the EDS microanalysis data (Figure S1) for the monolith–P. The spectrum of the Monolith–P sample does not contain any, while CRH-P-450 does contain less intense absorption bands at 2920–2840 cm^−1^, caused by the stretching vibrations of the C–H bond of aliphatic groups (methine CH–, methylene CH_2_–, and methyl CH_3_–). This indicates a higher degree of carbonization of the monolith-P compared to the CRH-P-450 component, which is clearly associated with the high-temperature monolith-P activation.

Thus, according to the FTIR spectroscopy data for the monolith–P, the component of which is modified with phosphate groups, the spectra clearly contain bands related to the vibrations of both oxygen-containing groups, 3400 cm^−1^—OH, 1620 cm^−1^—COO, and 1035 cm^−1^—C O-C, and phosphate groups, 1280 cm^−1^—P=O and 1160–1090 cm^−1^—P-O-C.

A broad absorption band in the region 3200–3600 cm^−1^ with the maximum around 3450 cm^−1^ of the PC-P sample’s FT-IR spectrum can be assigned to the O–H stretching mode of phosphoric acid functional groups. The band around 1220–1180 cm^−1^ can be attributed to the stretching of the P=O bond in a phosphate ester, the O–C bond in P–O–C linkage, or the P=OOH bond in phosphorus-containing groups. The band around 1700 cm^−1^ is usually caused by the stretching vibration of C=O in ketones, aldehydes, lactones and carboxyl groups; the band around 1600 cm^−1^ is attributed to an aromatic ring or C=C stretching vibration. The band at 1216 cm^−1^ is typically attributed to the C–O bond. The 1153 cm^−1^ band corresponds to the stretching vibration of hydrogen-bonded P=O and C–O stretching vibration in C–P, while the 1092 cm^−1^ band is related to P–O–P stretching [34].

### 2.3. Results of the Cytotoxicity Analysis of Monoliths

**Figure 10:**
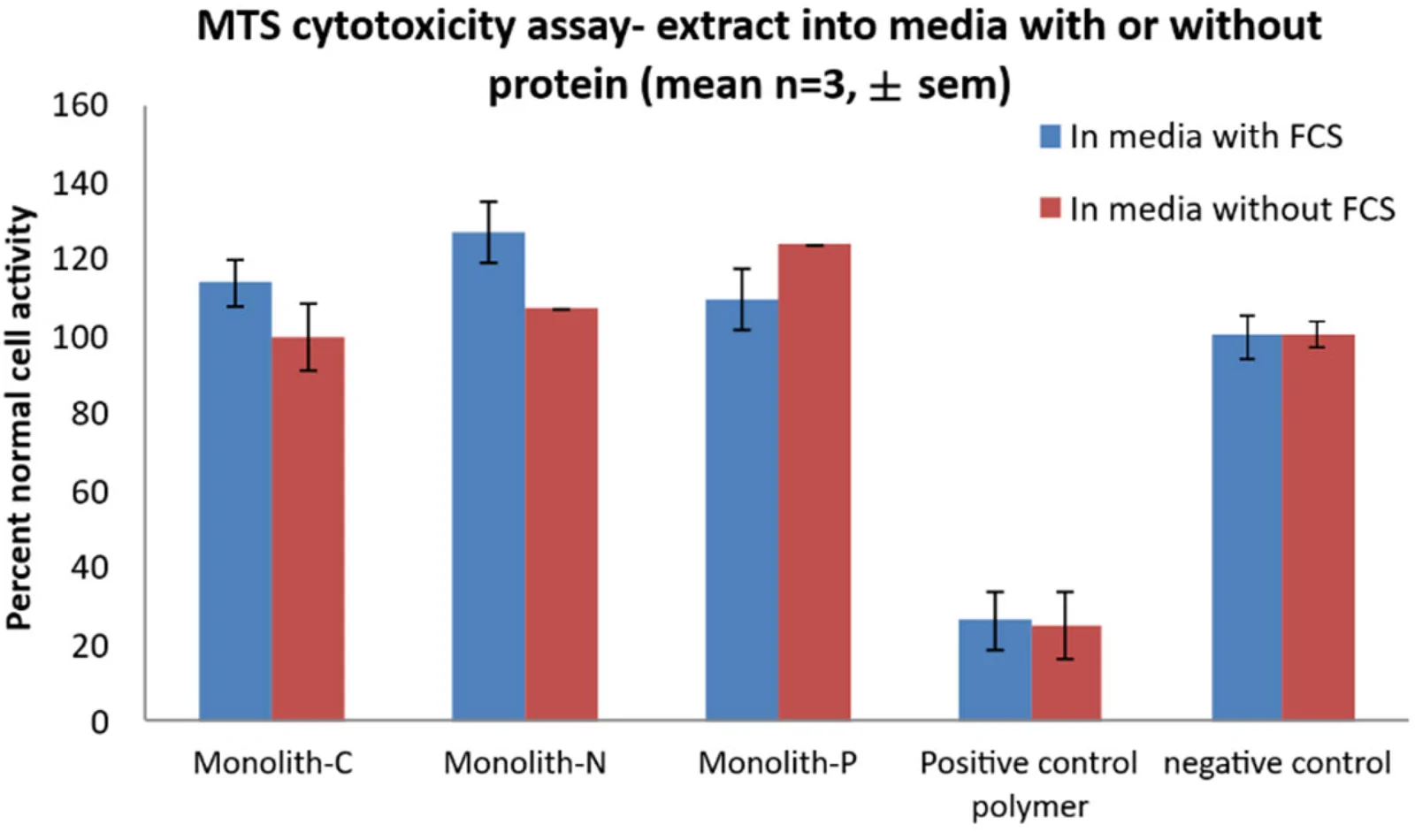
Percentage of normal cell viability measured using the MTS assay on 50% monolith extracts in media with or without FCS, compared to a positive control (mean 3 experiments (total *n* = 6 samples), ±sem).

**Figure 11:**
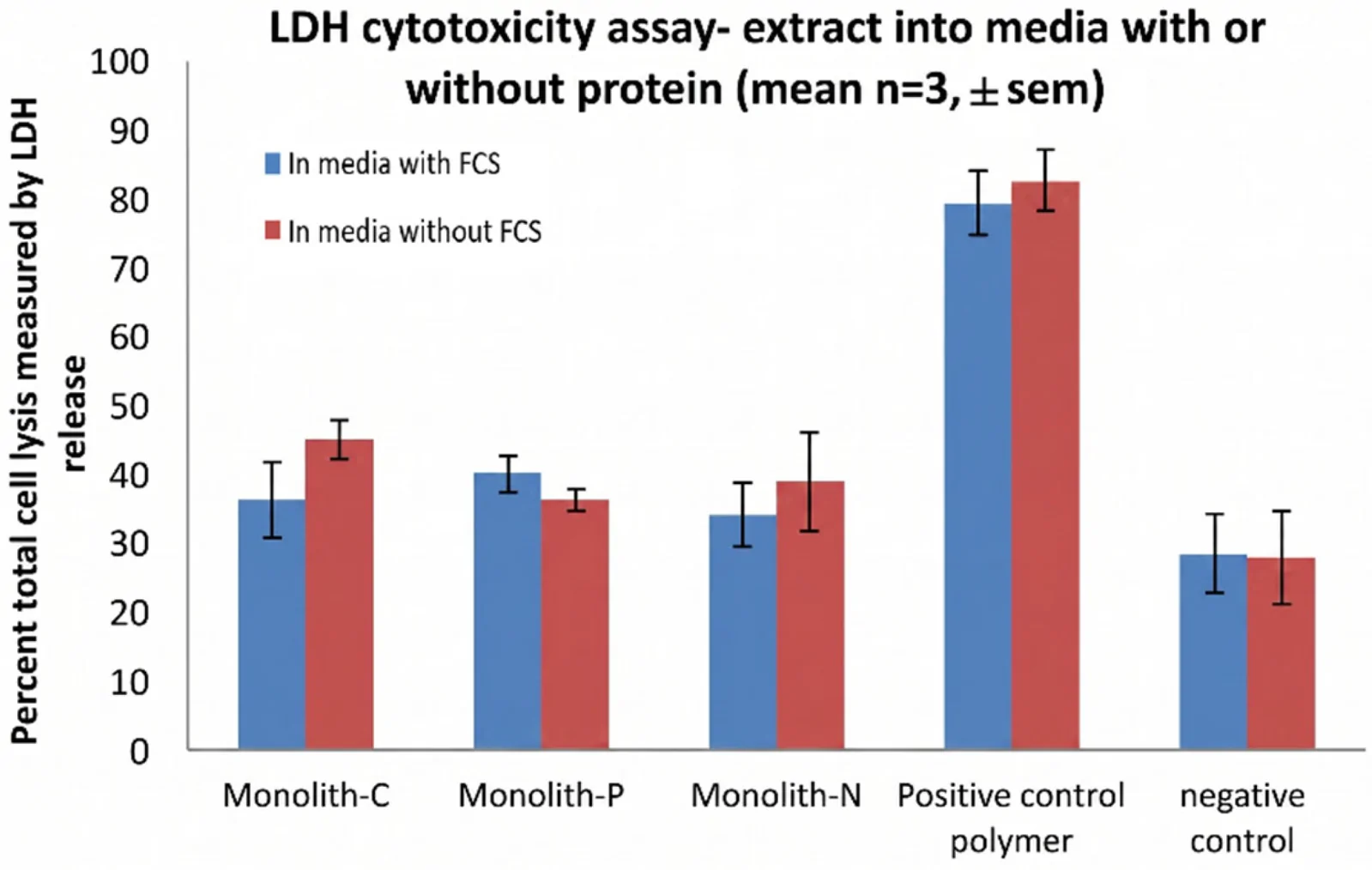
Percentage of cell lysis measured using the LDH assay on 50% monolith extracts in media with or without FCS, compared to a positive control (mean 3 experiments (total *n* = 6 samples), ±sem).

The 3-(4,5-dimethylthiazol-2-yl)-5-(3-carboxymethoxyphenyl)-2-(4-sulfenyl)-2H-tetrazolium (MTS) assay results (Figure 10) indicate that all three monoliths show percentages of normal cell viability comparable to the negative control (media only), and similarly, the lactase dehydrogenase (LDH) assay results (Figure 11) show percentage of cell lysis comparable to the negative control. There is no reduction in the percentage of normal cell viability or cell lysis between the monolith extracts into media with or without 50% extract of fetal calf serum (FCS) compared to the negative control (Student *t*-test *p* > 0.05). However, there is a significant difference between the values obtained for the monoliths in comparison with the positive control of the 50% polymer extract which showed only ~25% normal cell activity (*p* = 0.0002 and 0.0003 respectively in media with FCS) and ~85% cell lysis (*p* = 0.004, 0.002 respectively in media with FCS). The 50% extracts were used to assess the monolith cytotoxicity as the 100% extracts showed reduced viability and increased cell lysis compared to the negative control. This finding is in agreement with our previous study of the biocompatibility of phenol-formaldehyde-derived carbon honeycomb monoliths, which initially demonstrated an inhibition of colony formation and an apparent cytotoxic effect, but it was determined to be a result of nutrient depletion from the medium by adsorption due to the large surface area of activated carbon in comparison to the small surface area of the positive control polymer [35]. This starved the cells of nutrients and gave erroneous results. It has been acknowledged by other authors that alterations in culture media such as protein adsorption by test materials can influence the outcome of cytotoxicity evaluation and the need for precise standardization of testing procedures to encompass materials with high adsorption capacity [36]. Our findings for the 50% monolith extracts demonstrated that the monoliths did not cause any changes to the cell viability or cell lysis as measured by the MTS and LDH assay, and appear to be non-toxic to a V79 cell line under the conditions used and thus may be considered non-toxic to cells and warrant further investigation for hemoadsorption applications. As all three monolith types were deemed non-cytotoxic, the subsequent hemocompatibility investigation was carried out using the monolith-C, which exhibited the largest QSDFT surface area, making it a better candidate for hemoadsorption. Moreover, we have previously shown that N- and P-doped RH-derived nanostructured carbon adsorbents are non-toxic, according to MTS and LDH assay results [32].

### 2.4. Assessment of the Monolith-C Hemocompatibility

#### 2.4.1. Analysis of Platelet Activation

**Figure 12:**
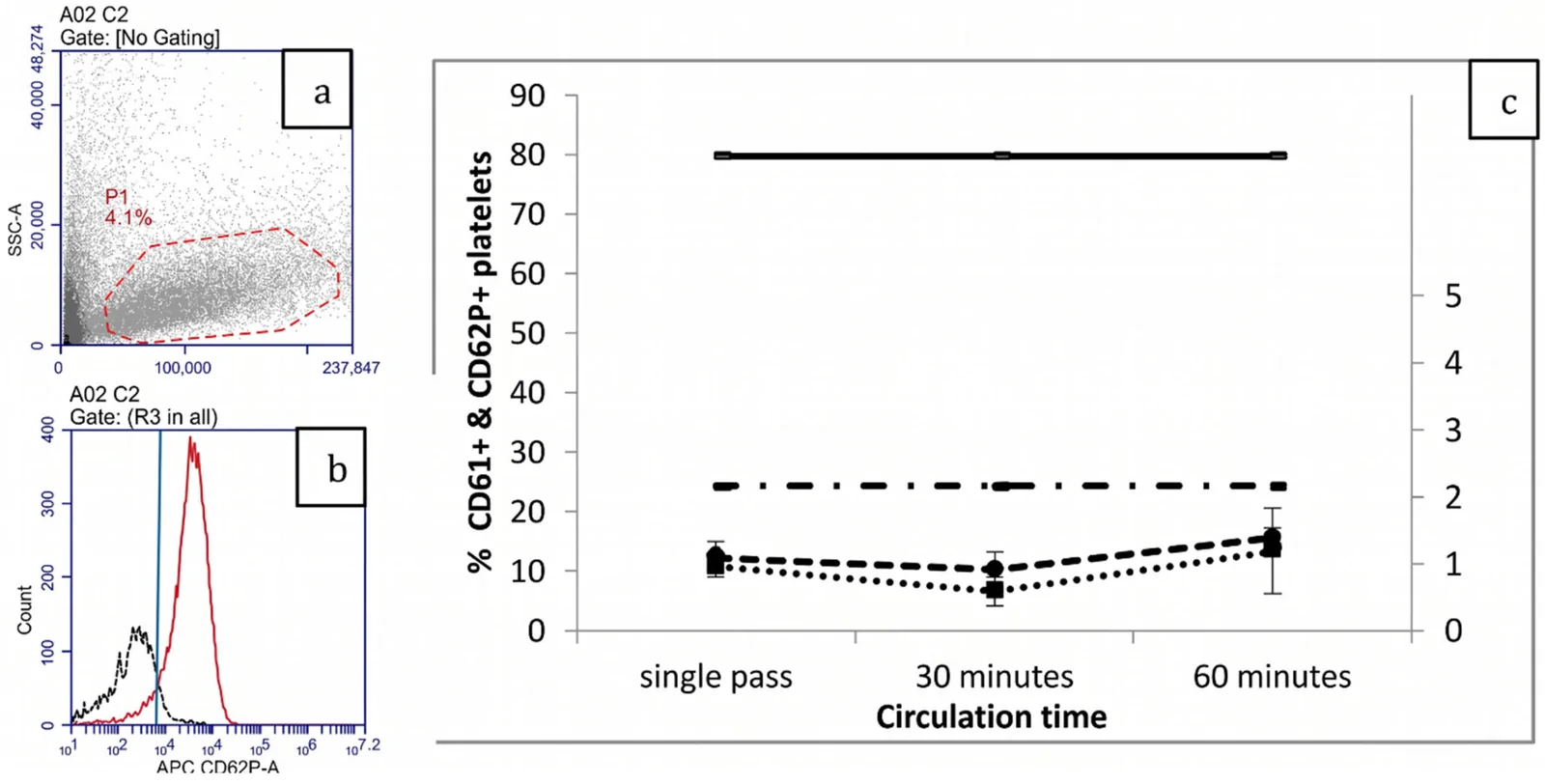
Flow dot plot gated (P1) on platelets (**a**) and histogram of the CD61+/CD62P+ platelets (**b**) for negative (black) and positive (red) samples. Graph (**c**) showing percentage of CD61+/CD62P+ platelets (activated platelets) for blood samples circulated through the monolith (- - -) or tubing-only control (**· · ·**) and negative (- **·** -) and positive (**—**) control samples measured by flow cytometry (mean *n* = 5 monoliths, ±sem).

**Figure 13:**
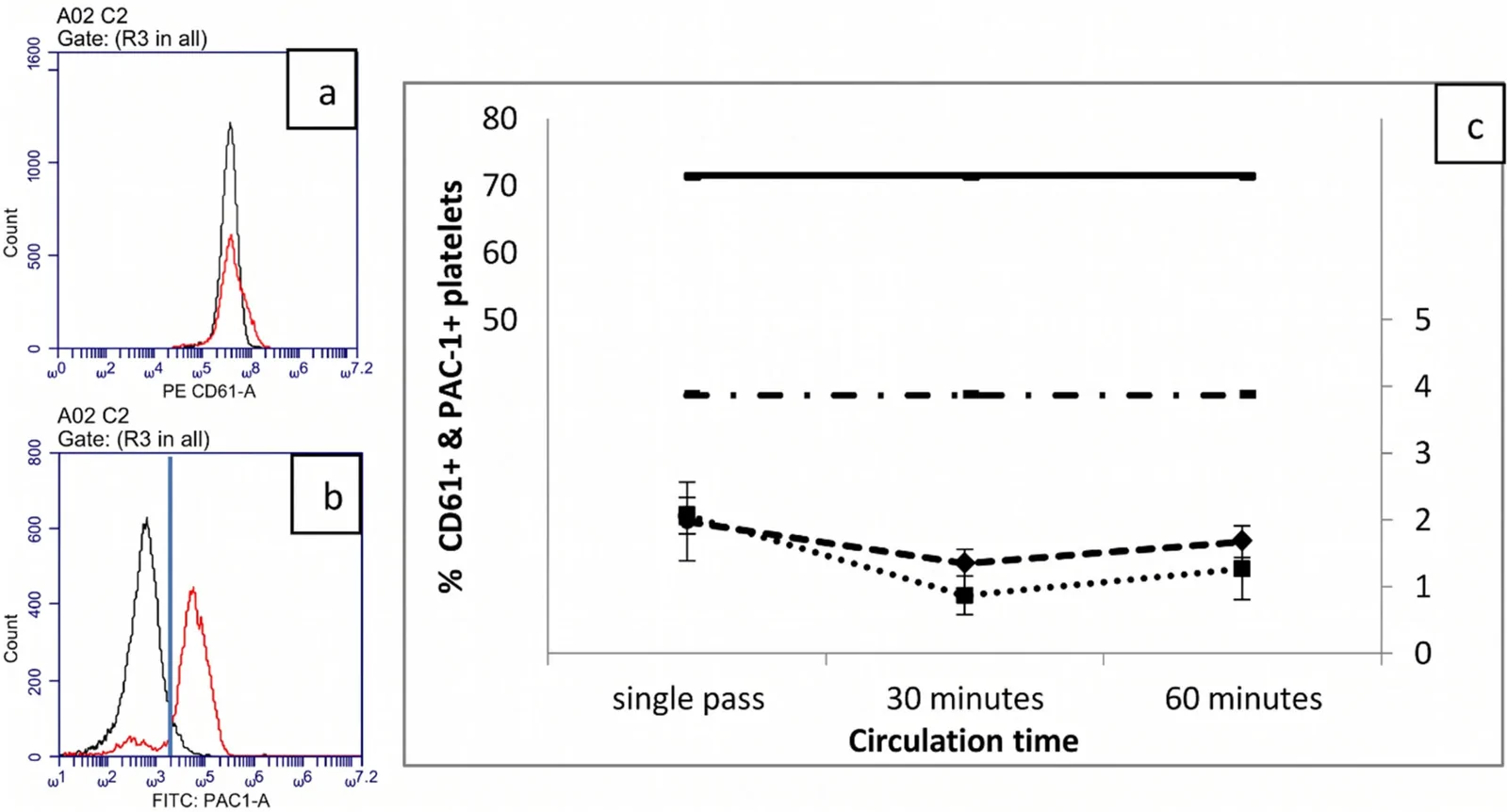
Flow cytometry histogram of gated CD 61+ platelets for both negative (black) and positive (red) samples (**a**) and histogram of the CD61+/PAC-1+ platelets (**b**) for negative (black) and positive (red) samples. Graph (**c**) showing percentage of CD61+/PAC-1+ platelets (activated platelets) for blood samples circulated through the monolith (- - -) or tubing-only control (**· · ·**) and negative (- **·** -) and positive (**—**) control samples measured by flow cytometry (mean *n* = 5 monoliths, ±sem).

Differentiation of the platelet population in the blood samples, using flow cytometry by gating on the platelet specific membrane glycoprotein GpIIIa (CD61), allowed a distinction between non-activated platelets and those which were activated using the activation markers: anti-activated glycoprotein 11b/11a fibrinogen binding site marker (PAC1) and p-selectin (CD62P). The results (Figure 12 and Figure 13) demonstrate that the assay was able to distinguish between platelets that are resting (negative control) with lower fluorescence levels for activation markers CD62P (Figure 12c) and PAC-1 (Figure 12c) and those that have been activated with a positive control of blood activated by phorbol myristate acetate (PMA), showing noticeably higher fluorescence levels for these two activation markers. By gating on the fluorescence histograms of PAC-1 and CD62P for the CD61 positive platelet population, the percentage of PAC-1 (Figure 12b) and CD62P (Figure 13b) positive platelets (activated) were determined. There was no appreciable platelet activation in blood samples circulated through the monolith-C or tubing-only control over the 60 min duration of the experiment, suggesting that the monolith-C is not activating platelets to any great degree and thus could be considered hemocompatibility. These results follow findings from other studies which demonstrated high protein adhesion on monolith-C surfaces, although they inferred that it was unlikely that the initial protein layer would be replaced by any platelet-derived adhesive proteins, thus limiting inflammatory cell adhesion [37]. Similarly in our previous biocompatibility study with phenol-formaldehyde-derived activated carbon wells [38], although some platelet aggregation was seen at the surface, no highly activated platelets as evidenced by granule release were observed.

#### 2.4.2. Analysis of Granulocyte and Monocyte Activation

**Figure 14:**
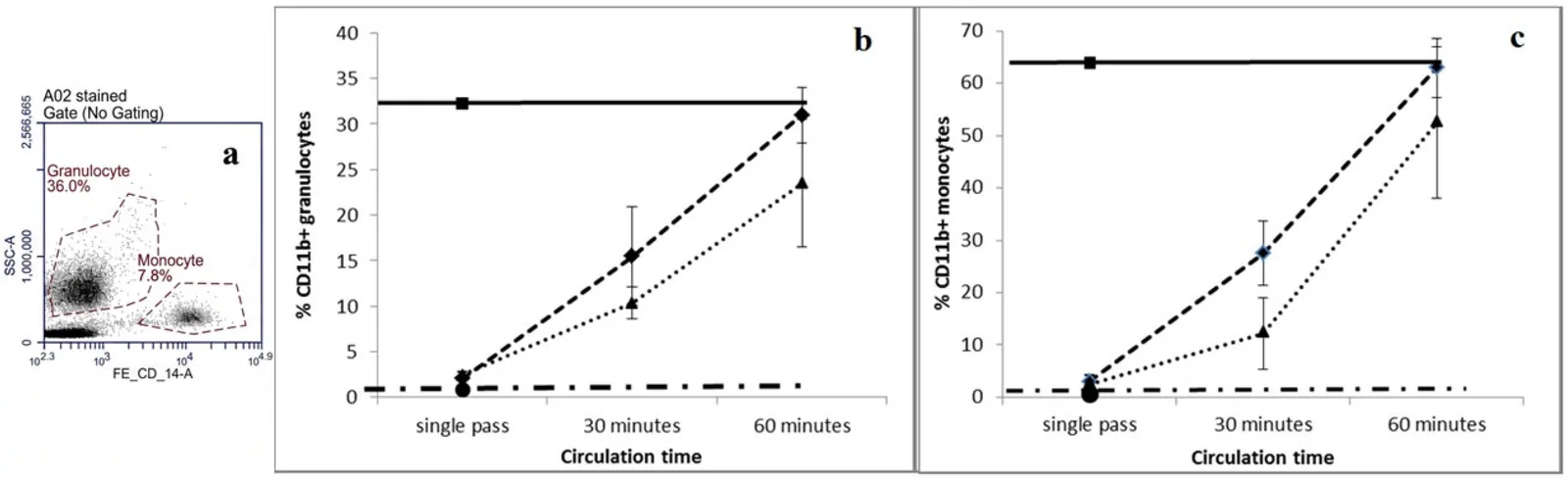
Flow dot plot gated on granulocytes and monocytes (**a**). Percentage of CD11b+ granulocytes (**b**) and CD11b+ monocytes (**c**) for blood samples circulated through the monolith (- - -) or tubing-only control (· · ·) and negative (- · -) and positive (**—**) control samples measured by flow cytometry (mean *n* = 5 monoliths, ±sem).

Identification of the granulocyte and monocyte populations within the blood samples using flow cytometry was accomplished using the differential expression of the membrane glycoprotein CD14, followed by activated cells distinguished using the activation marker CD11b. The results in Figure 14 show the percentage of CD11b+ granulocytes and monocytes (activated cells) for blood samples circulated through the monolith or tubing control, showing an increase in activation over time compared to the negative control at time zero. It is clear that in the case of the silicone tubing system control, there was appreciable activation of both cell types during circulation through the roller pump over the 60 min experiment. However, there was no significant difference in the levels of granulocyte and monocyte activation within the system upon the addition of the monolith even at 60 min (two-way ANOVA, *p* = 0.65, 0.89 respectively). Other in vitro studies have shown similar granulocyte activation in blood using both centrifugal and roller pumps, indicated by a significant increase in the granulocyte activation proteins, calprotectin, lactoferrin and myeloperoxidase, with the elevation occurring earlier with the roller pump [39]. It is known that the use of roller pumps in cardiopulmonary bypass surgery is one of the more important factors contributing to the recorded adverse effects, such as an inflammatory response [40]. In our previous batch experiments with AC wells, only moderate granulocyte and monocyte adhesion to the surface was demonstrated, and no evidence of degranulation by activated granulocytes measured by chemiluminescence was detected [38]. Testing the monolith hemocompatibility in this dynamic test system was chosen to best mimic a standard hemodialysis session; however, it is clear that the use of a roller pump interferes with the assessment of blood cell activation, and future studies may benefit from the use of a centrifugal pump.

#### 2.4.3. Assessment of Coagulation Parameters

The coagulation kit Unicalibrator, and N and P controls for all four tests conducted fell within the ranges recommended by the manufacturer. The coagulation results for PPT, PT and Thrombin generation times showed no difference in the clotting times between the monolith and tubing controls, and the negative control results at time zero. The results presented in Table 2 are shown for the blood plasma of one blood donor; the same pattern of clotting times was repeated in the other two experiments, but the negative control values varied between donors. The clotting times all fell within the normal range for healthy patient plasma and showed no significant difference between the tubing-only control and monolith, as calculated by the two-way ANOVA (APPT *p* = 0.55, PT *p* = 0.95, Thrombin *p* = 0.38). Measurement of the PPT clotting time highlights the intrinsic pathway which can be activated by ‘foreign surfaces’ in direct contact with blood and indicates changes in the presence and activity of factors XII, XI and IX. The PT clotting time is an indicator of the extrinsic and common coagulation activation pathway [41] and the TT provides information about the main coagulation reaction, the transformation of fibrinogen into fibrin. An increase in either of these clotting tests would suggest that the monolith had either adsorbed some of the pathway factors resulting in an enhanced anti-coagulant activity, or they had been depleted during activation of the coagulation cascade upon contact, leading to thrombus formation [42,43]. Overall, our findings suggest that the activated carbon monolith Made of rice husk does not activate either the intrinsic or extrinsic clotting pathways, and thus their use in an extracorporeal device would not increase the risk of thrombus formation within the device. The results for the fibrinogen concentration in the samples show no difference between the monolith and tubing and the negative control results at time zero, with no significant difference between the tubing-only control and monolith (two-way ANOVA, *p* = 0.82). The levels of fibrinogen (g/L) calculated using the STart^®^ Hemostasis benchtop Analyzer were higher than the normal levels found in plasma (1.5–3 g/L), although the Fibri-prest kit controls matched the recommended values. This anomaly may be due to the inbuilt calibration calculation for the fibrinogen standard curve and should not negate the fact that the fibrinogen levels between the negative controls and samples were similar. These findings suggest that there is no significant loss of fibrinogen by adsorption to the monolith, which is a positive result in the context of material hemocompatibility as fibrinogen adsorption is required for platelet adhesion to the surface of a material and subsequent activation [36]. It is known from other hemocompatibility studies that 3D porous carbon exhibit lower fibrinogen/albumin adsorption ratio due to the material’s hydrophobicity, correlating with a reduction in platelet adhesion and improved thromboresistance of materials [44].

#### 2.4.4. Assessment of Hemolysis

Hemolysis testing is an important component of assessing hemocompatibility of materials as it can be triggered not only by the mechanical effect of sheer stress on blood during circulation through the pump and device, but also by the release of factors from activated platelets and white blood cells. It has been recognized that plasma Hg is a biologically hazardous molecule, mainly due to its iron-mediated oxidizing capabilities [45,46]. The results presented here indicate that the circulation process through the roller pump caused some hemolysis of the blood, as the plasma free Hg level measured pre-study (25.7 mg/dL) was much lower than even after the single passage through the monolith or tubing control (39.5 vs. 55.3 mg/dL) and further increased by 60 min circulation (64.4 vs. 106.5 mg/dL). This is not unexpected as it is known that flow rate can have a major effect on hemolysis and tests, with a conventional roller pump used in routine cardiopulmonary bypass in infants and children that was shown to significantly elevate free Hg levels (~70 mg/dL post bypass) to values similar to our study [47]. In a similar CPB study it was demonstrated that a centrifugal pump caused less hemolysis than a roller pump [48]. While our study was limited by the use of a roller pump, the results show that the monolith does not cause any additional increase in free Hg, and thus, hemolysis was comparable to the tubing-only control. It is possible that the lower Hg levels seen with the monolith may be in part due to adsorption of the generated Hg molecules by the monolith; however, a previous study on autologous blood transfusion had shown that unmodified activated carbon had a low capacity for Hg and required surface modification with polypyrrole to increase the adsorption rate of free hemoglobin from aqueous suspensions [49].

#### 2.4.5. Assessment of Complement Activation

**Figure 15:**
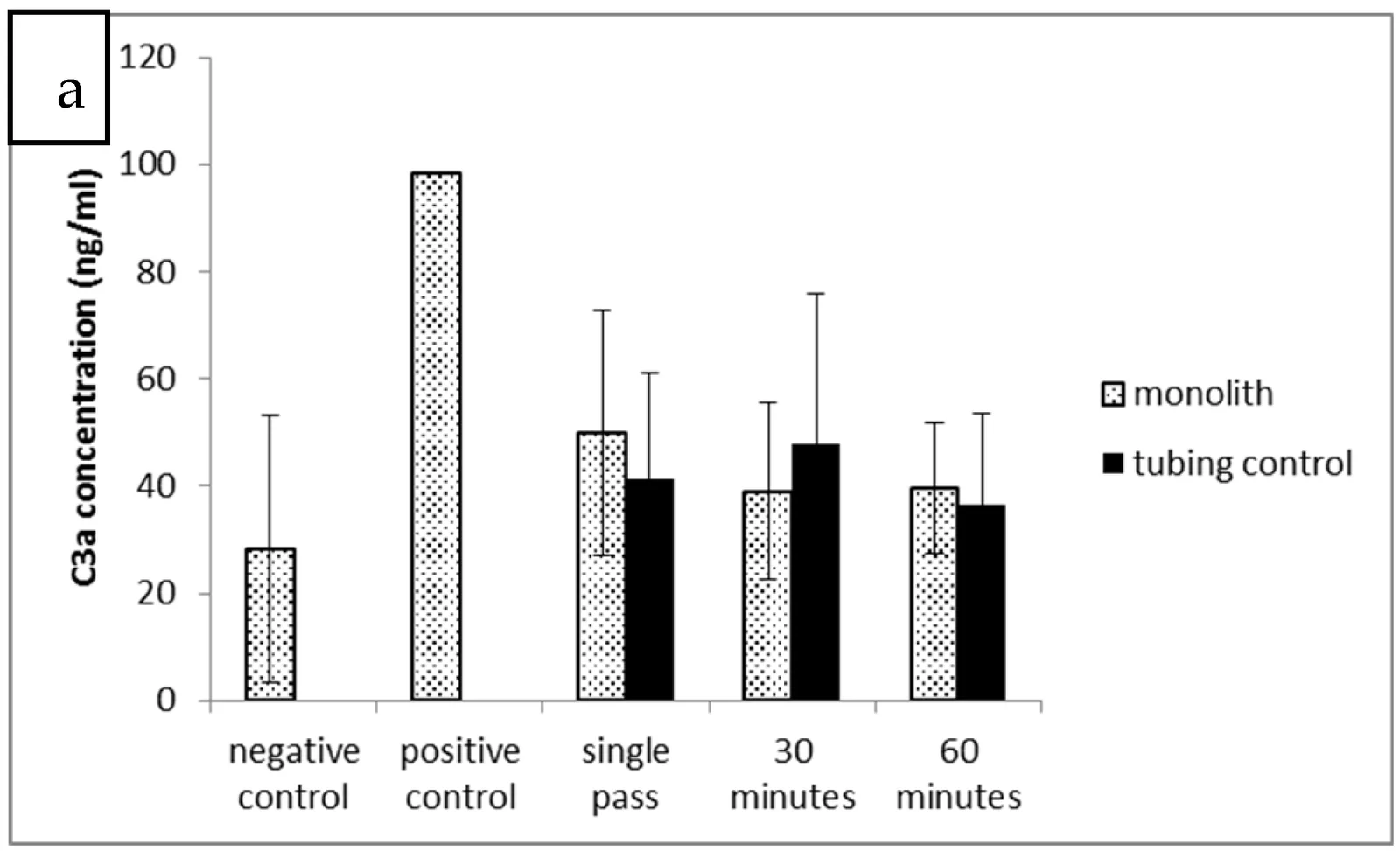

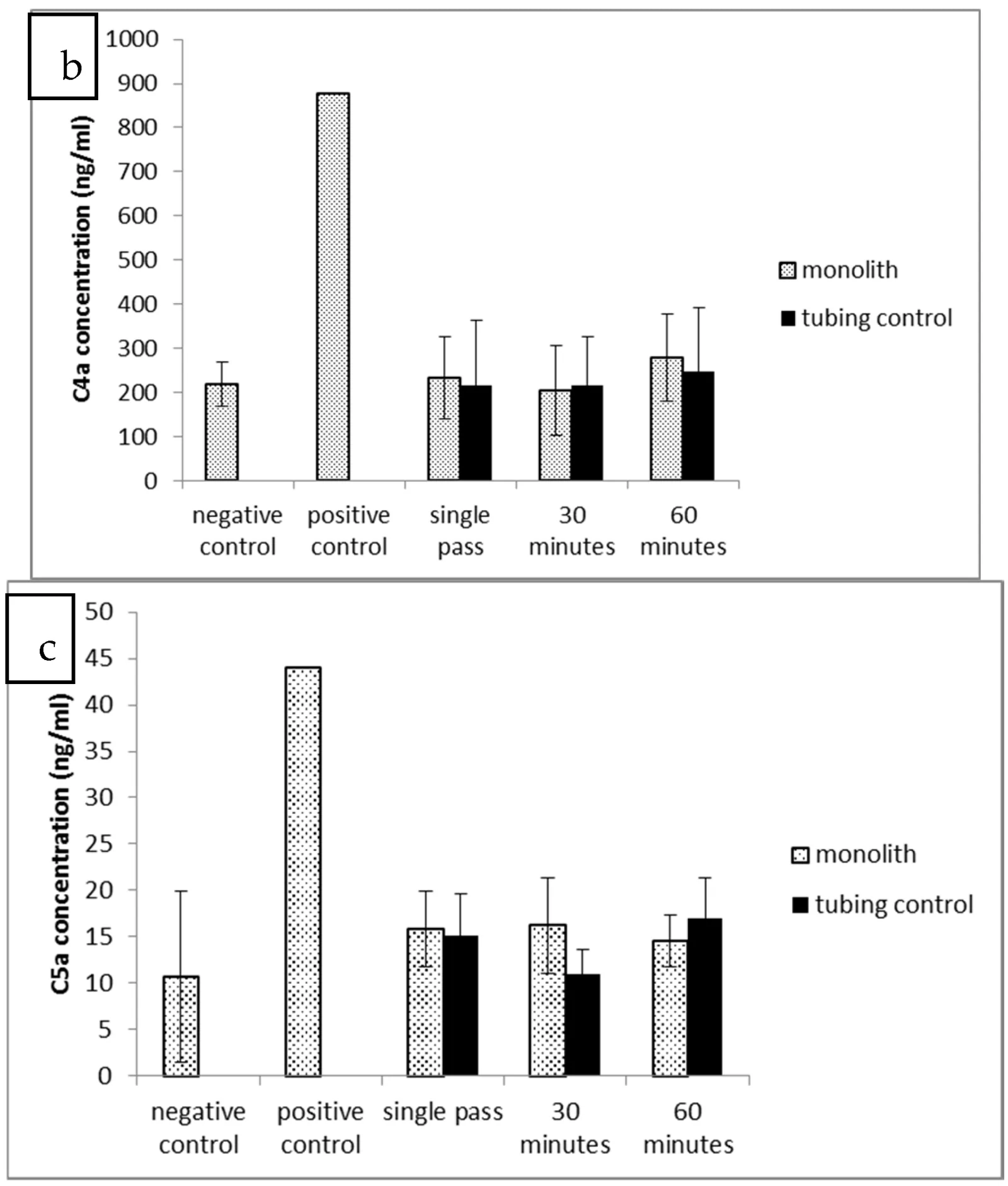
Complement C3a (**a**), C4a (**b**), and C5a (**c**) concentration (ng/mL) in blood samples after the circulating experiment through the monoliths and tubing control, compared to negative control at time = 0 and positive control activated with Zymosan A (mean *n* = 5 monoliths, ±sem).

The C3a, C4a and C5a complement activation results (Figure 15) for the recirculating experiments after a single pass, 30 min and 60 min showed no significant difference in the concentrations measured between the monolith and tubing controls (two-way ANOVA, *p* = 0.92, 0.99 and 0.66 respectively) and the negative control at time zero (*t*-test, negative and single pass *p* > 0.5). The concentrations of the three complement components are comparable to those recorded in the literature for healthy donors at time zero and, in particular, for samples stabilized by the use of Futhan [50,51]. In addition, concentrations of all three components were lower than the positive control with Zymosan A, which is an activator of the alternative pathway relevant for both C3a and C5a, although it can also activate the classical pathway via anti-yeast antibodies [52]. The data were taken from three separate blood donor experiments with two monoliths and single controls for each experiment; as a consequence, there was variation in the initial donor complement levels as evidenced by the relatively large standard deviations measured. However, these initial findings suggest that the monoliths do not activate the complement components C3a, C4a, and C5a to any great extent. When evaluating complement activation by such a powerful adsorbent as activated carbon, the results may be affected by the ability of the monolith-C to adsorb the components generated by the activation of either complement pathway. Therefore, there is potential for monolith-C to mask the degree of complement activation. In our previous study we measured low C3a and iC3b levels generated by phenol-formaldehyde-derived activated carbon wells, which suggested a low degree of complement activation; however, further analysis revealed that some iC3b was adsorbed onto the monolith-C surface [38]. Other studies with the dialysis membrane polyacrylonitrile (PAN) have also shown the ability to adsorb C3a from the aqueous phase [53]. Overall, the complement activation findings for the monolith indicate that there is no excessive complement activation, and any complement that is generated could be adsorbed by the monolith-C surface, thus minimizing its contribution to the inflammatory response.

### 2.5. Assessment of Protein-Bound Uremic Toxins and Inflammatory Cytokines Adsorption Using Functional Nanostructured Carbon Honeycomb Monoliths

The adsorption characteristics of carbon monoliths prepared from rice husk were assessed for their removal efficiency for a range of biological toxins in a recirculation system using fresh frozen human plasma to mimic the process of hemoperfusion in the clinic.

The biological toxins chosen to be investigated were the inflammatory cytokines, IL-6 and IL-8, as these molecules cover a range of sizes from 8 to 51 kDa and are involved in a number of clinical conditions where there is a dysregulated inflammatory response, for example, in sepsis. A second set of toxins chosen for investigation were the uremic toxins, p-cresyl sulphate and indoxyl sulphate; these are relatively small molecules, but they are protein-bound in the blood, and thus these molecules are hard to remove using current extracorporeal therapies such as hemodialysis in patients with renal failure.

**Figure 16:**
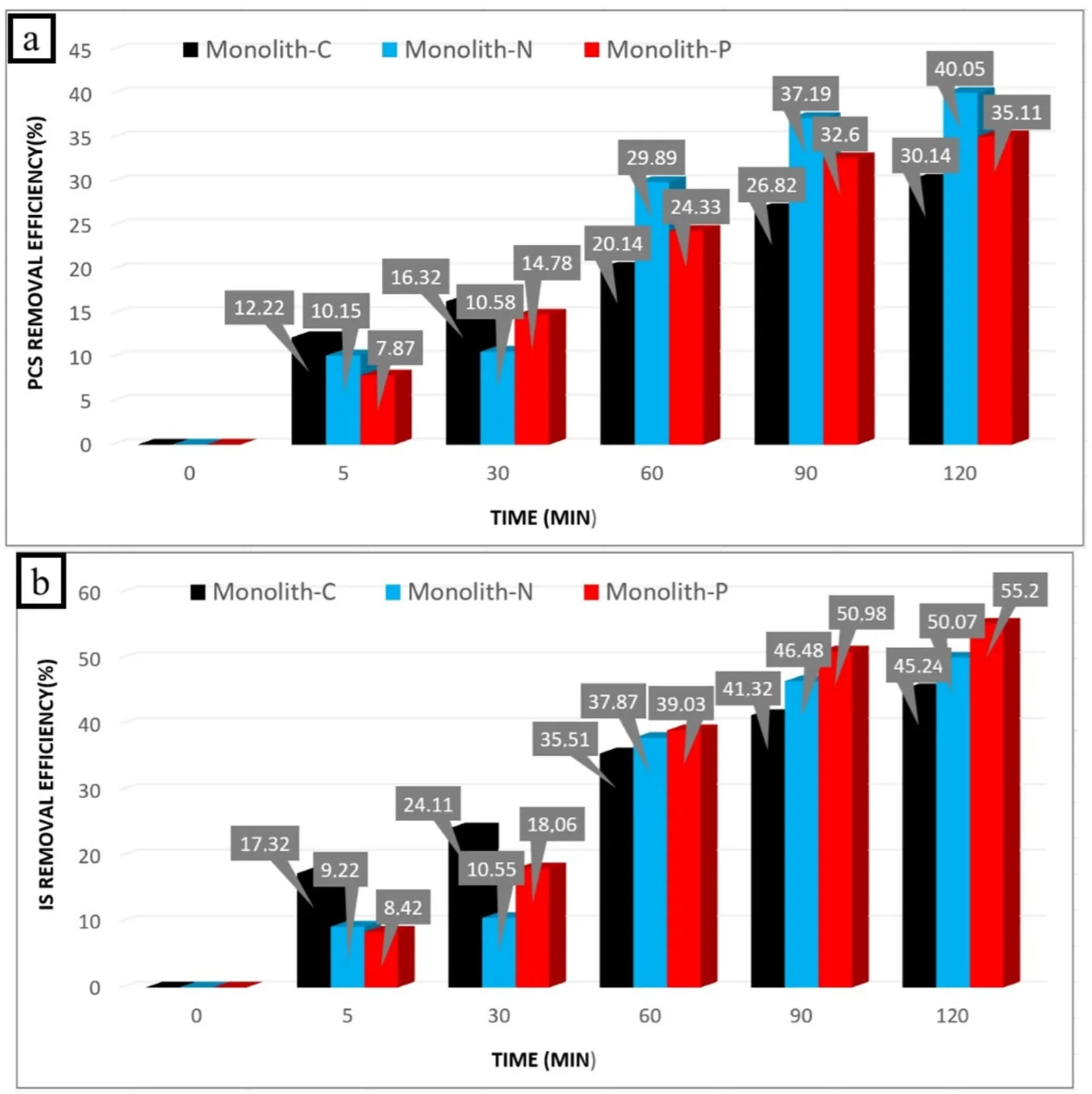
PCS (**a**) and IS (**b**) adsorption profiles for monoliths, as well as removal efficiency increases during 2 h of recirculation in human plasma.

The adsorption profiles for three monoliths and removal efficiency of PCS and IS during 2 h of recirculation in human plasma are shown in Figure 16 (Figure S3a,b).

From Figure 16 and Table 3 it follows that after 120 min of plasma recirculation, the monolith-N, which has anion-exchange properties, exhibited a higher PCS removal efficiency (ca. 40%), compared to the monolith-C (ca. 30%) and monolith-P (ca. 35%), despite having the smallest mesopore BJH volume. On the other hand, the monolith-P, which has cation-exchange properties and has the largest mesopore volume, exhibited a higher IS removal efficiency (ca. 55%), compared to the monolith-N (ca. 50%) and monolith-C (ca. 45%). Protein-bound PCS and IS adsorption profiles for the monoliths over 2 h recirculation in human plasma are shown in Table S2.

**Figure 17:**
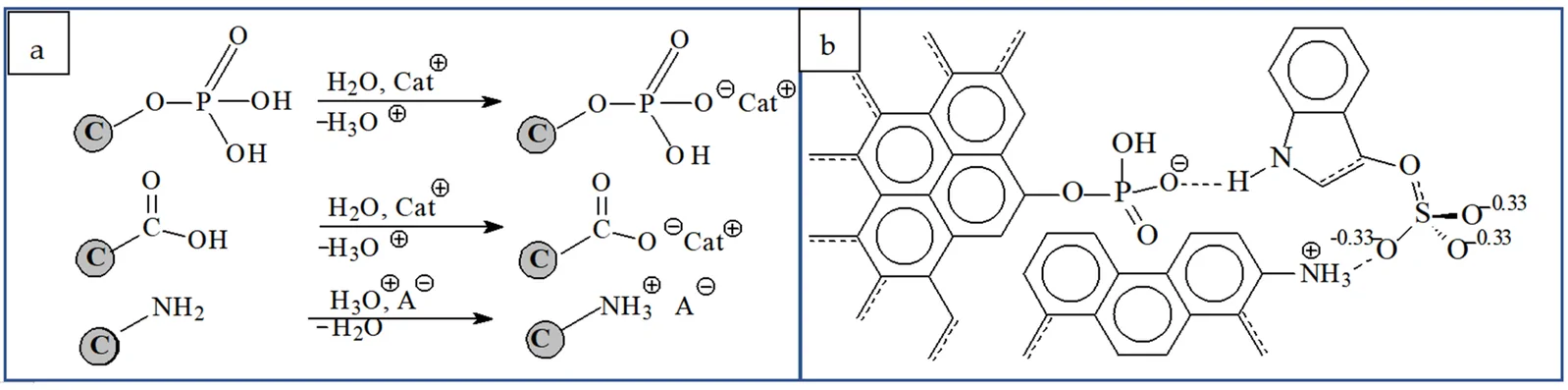
Interaction schemes of: (**a**) cation-exchange (Cat^+^) phosphate, carboxylic groups and anion-exchange (A^−^) amino groups with respective ionic groups of a carbon substrate; (**b**) surface phosphate and amino groups with indoxyl sulphate molecule.

A typical idealized scheme of interaction mechanism between surface functional ion-exchange groups and corresponding ionic groups of a functionalized carbon monolith surface are present in Figure 17. Plausible mechanisms of interaction (due to ionic interaction or hydrogen bond formation) for amino groups with sulphate groups of IS and PCS, as well as phosphate (or carboxylic) groups with N-H groups of pyrrole ring in IS, are illustrated in Figure 17b.

Apparently, the cation–anion-exchange amino groups grafted onto the carbon surface play a positive role in the removal efficiency of the monoliths.

**Figure 18:**
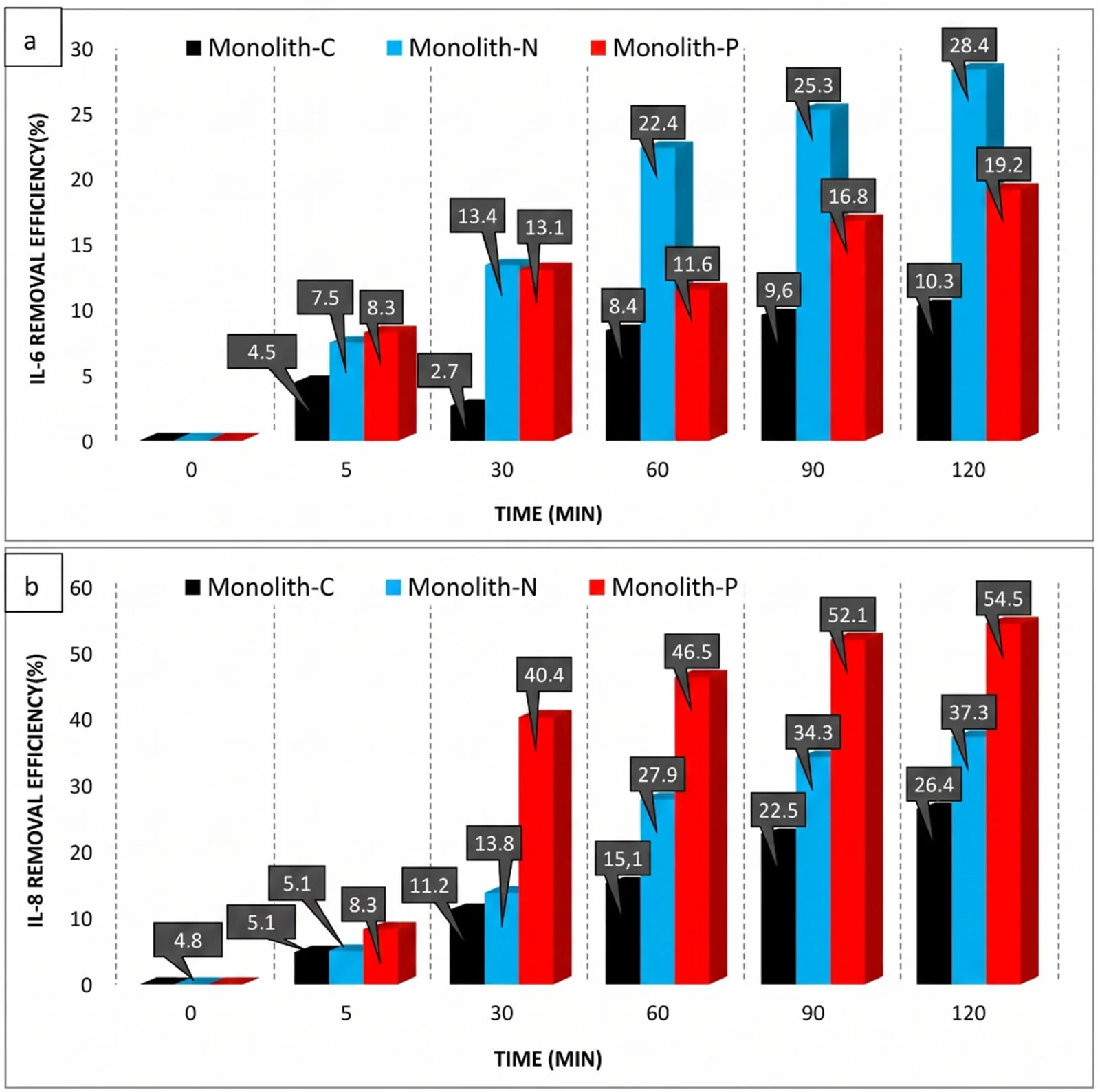
IL-6 (**a**) and IL-8 (**b**) adsorption profiles for monoliths: removal efficiency increases during 2 h of incubation in human plasma.

The adsorption profiles for the monoliths and adsorption capacities of IL-6 and IL-8 during 120 min of recirculation in human plasma are shown in Figure 18 and Figure S3c,d, and Table S2.

From the data in Table 3, it follows that after 120 min of plasma recirculation, the Monolith-P, which has cation-exchange properties and has the largest mesopore volume, showed a higher degree of IS adsorption (ca. 55%), compared to the Monolith-N (ca. 50%) and Monolith-C (45%). The IL-6 removal efficiency using the Monolith-N exceeds that of the Monolith-C, despite its smaller mesopore volume. Apparently, the anion-exchange amino groups grafted onto the carbon surface play a positive role in the removal efficiency.

The Monolith-N exhibits superior IL-8 sorption compared to the Monolith-C, despite its smaller mesopore volume. Apparently, the anion-exchange amino groups grafted onto the carbon surface play a positive role in the sorption capacity for this cytokine. Based on the results of adsorption studies under dynamic conditions, it was revealed that monoliths modified with ion-exchange groups are capable of eliminating clinically significant levels of proinflammatory cytokines from blood plasma and exhibit better sorption capacity compared to the unmodified Monolith-C. Despite the fact that the Monolith-P modified with phosphate groups exceeds the Monolith-N in mesopore volume by more than two times, its removal efficiency under dynamic recirculation conditions in relation to the toxins IS and IL-6 is somewhat inferior. Textural characteristics such as pore volumes and specific surface areas are not determining factors in assessing removal efficiency under dynamic recirculation conditions for specific toxins; surface chemistry also plays a role. By varying the textural parameters of the sorbents, as well as the composition of the ion-exchange functional groups on the carbon surface, it is possible to impart selectivity to the resulting modified adsorbents in relation to the toxins removed. Based on the results of adsorption studies under dynamic conditions, it was revealed that modified samples are capable of eliminating clinically significant levels of albumin-bound azotemic toxins from blood plasma and exhibit better sorption capacity compared to the unmodified Monolith-C.

In the previous study, the patterns observed for the nanostructured carbon adsorbents PC-N1 and PC-P (CRH-P-450) in the adsorption of low-molecular-weight organic compounds were largely preserved for the corresponding monoliths [32]. This indicates that the formation of the monolithic structure did not lead to a significant loss of accessibility of active sites and did not change the dominant adsorption mechanism. The main differences between Monolith-N and Monolith-P remain determined by the combination of their porous structure and surface chemistry inherited from the original powder precursors. If we compare these data with the previously obtained characteristics of the nanostructured functionalized carbon adsorbents, we can propose that:

- Monolith-N (analogous to ammoxidized PC-N1) retains an advantage in the adsorption of molecules for which narrow microporosity and π–π/Lewis interactions are critical (IL-6 and PCS). This corresponds to a high proportion of narrow micropores and the presence of nitrogen-containing functional groups.
- Monolith-P (derived from PC-P) shows better results for IL-8 and IS, which is likely due to the greater role of surface chemistry—primarily oxygen- and phosphorus-containing functional groups that can enhance electrostatic interactions and hydrogen bond formation with these adsorbates.

The adsorption behavior of IL-6 and IL-8 on monoliths can be partially explained by differences in their total surface charge at physiological pH 7.4. IL-6 (pI ≈ 5.98) has an overall negative charge, whereas IL-8 (pI ≈ 8.11) has a positive charge [54,55,56,57,58]. Representative residues, assigned according to the canonical amino acid sequences of human IL-6 (UniProt accession P05231) and IL-8 (UniProt accession P10145), are presented in Table 4 and Table 5, (Figure S4 and Figure S5) respectively.

Recent studies have shown that positively charged cytokines exhibit stronger electrostatic interactions with negatively charged biological matrices due to their affinity for anionic glycosaminoglycans. Although these observations were reported for extracellular matrix components and not for carbon adsorbents, they support the general concept that electrostatic interactions may facilitate cytokine adsorption. The authors cite IL-8 as an example of a cytokine that binds to heparin via positively charged surface regions. Thus, there is experimental evidence that IL-8 actively participates in electrostatic binding [55]. Although electrostatic interactions contribute to adsorption, the observed adsorption trends cannot be explained solely by the total surface charge or pHpzc. For example, the undoped carbon monolith did not exhibit adsorption characteristics intermediate between nitrogen- and phosphorus-doped samples. This suggests that adsorption is determined by a combination of pore architecture, adsorption site accessibility, aromatic interactions, and the nature and spatial distribution of surface functional groups, rather than by the total surface charge alone.

Thus, for the monoliths studied, there is a hierarchy of factors rather than a single dominant mechanism. One of the main factors is pore accessibility and the appropriateness of pore size to molecular size of an adsorbate, especially for cytokines. Selectivity of interaction, in our case, is determined by the type and distribution of functional groups, including N- or P-containing centers. For aromatic uremic toxins (PCS and IS), π–π and hydrophobic interactions make major contributions. Electrostatic interaction should be considered as a factor modifying the already established affinity between the sorbate and the sorbent surface.

A good hemoperfusion adsorbent needs to meet the following criteria: (1) strong adsorption capacity and high selective adsorption efficiency; (2) high specific surface area, high porosity, and suitable pore size distribution; (3) little influence on the components of blood, especially the loss of blood cells and macromolecular proteins, no hemolysis, and no coagulation; (4) good biocompatibility, nontoxicity, and incapacity to stimulate human inflammatory responses; (5) stable physical and chemical properties, resistance to decomposition, deformation, and degeneration; (6) specific mechanical strength to withstand the flow friction in the perfusion process and resistance to breaking; (7) convenient disinfection and storage; and (8) simple preparation process suitable for large-scale production and low cost [27].

The results of our team’s preliminary yet extensive studies have been able to demonstrate that the functional nanostructured carbon honeycomb monoliths, derived from low-cost renewable biomass using “green” technology approaches and designated for hemoadsorption, are biocompatible, i.e., hemocompatible (with laminar blood flow along the monoliths’ channels), unlike the granular ACs, which cause wobbling, thus causing blood-forming elements damage; therefore, they show no sign of cytotoxicity; the results also demonstrate that the monoliths are suitable for protein-bound uremic toxins and inflammatory cytokine elimination via the HA technique, and thus the monoliths do meet most of the aforementioned requirement criteria. They are also selective due to their surface tailoring with amphiphilic surface ion-exchange functional groups, which presume not only physisorption, but also chemisorption.

## 3. Materials and Methods

### 3.1. Preparation of Monoliths-C

The carbon monolith was prepared as follows: Washed, dried and milled rice husk (RH) (particle size fraction: 40–63 μm) was mixed with organosolv lignin as a binder using a Z-blade mixer at a mass ratio of 75:25 respectively. The resulting RH-lignin paste was extruded through a 600 CPI 27 mm diameter die plate to produce the “green” monoliths (Figures S6 and S7). These “green” monoliths were subsequently carbonized at 973 K for 10 min in a large pot furnace under a CO_2_ flow of 3 L/min, with a heating rate of 1 K/min from room temperature to 973 K. Thereafter, the carbonized monoliths were activated at 1123 K for 3 h under CO_2_ flow in a horizontal tube furnace (Figure S8). The total mass loss after carbonization and activation was approximately 65%. Finally, the activated monoliths underwent SiO_2_-extraction, being digested with 1.5 M NaOH at 353 K for 5 h, thoroughly rinsed with deionized water to remove residual sodium silicate, and then dried at room temperature for 30 h until constant weight was achieved. The weight loss after SiO_2_ extraction was around 30% relative to the activated monolith. Overall, this corresponds to a total mass loss of approximately 75.5% relative to the initial green monolith.

Honeycomb carbon monoliths-C were produced from rice husk powder (RH) and a lignin-based organic carbonizable binder. The RH/binder ratio of 3:1 was found to provide the best balance between strength and pore structure retention. The extruded monoliths were heated to 700 °C at a temperature increase rate of 1 °C/min and carbonized for 10 min in CO_2_. For subsequent flow experiments, the carbon monoliths, with an average weight of 1.13 g, were heat-shrink-wrapped with polyolefin heat-shrink tubing (RS Components Ltd., Corby, UK) [59].

### 3.2. Preparation of Monoliths-N

Carbon monoliths (Monolith-C), obtained from rice husk and lignin, were subjected to ammoxidation. The monoliths were placed in ceramic boats and introduced into a flow-through quartz reactor located in a horizontal electric furnace. To generate ozonides and other oxygen-containing surface groups, the samples were first treated with an ozone–air mixture (1 L min^−1^) at 130 °C for 6 h. Immediately after the ozonation step, ammonia was introduced into the reactor at a flow rate of 5–6 mL min^−1^. The temperature was then increased to 350 °C and maintained for 3 h. To verify that nitrogen was chemically bound to the carbon matrix, monolith-N was treated with concentrated HCl for 5 min, followed by washing with distilled water until neutral pH was reached to yield the monolith-N_HCl.

### 3.3. Preparation of Monoliths-P

The molding compound was prepared from 112.5 g of ground CRH-P-450 (fraction less than 70 µm) and a binder of the following composition: lignin—37.5 g; methylcellulose—20.5 g; polyethylene oxide—1.5 g; and water—262.5 g. The molding compound was mixed in a Z-shaped mixer, extruded through a die (cell density—36 cpi, diameter—10.5 mm) (Figures S7 and S8), and cured in a drying oven. To obtain monolith-P samples, honeycomb extrudates were heated at a temperature rise rate of 1 °C/min to 500 °C and carbonized for 1 h.

A schematic diagram of a setup for producing micro-mesoporous carbon materials, including honeycomb sorbents/carriers, was developed. A drawing of the setup for carbonization and activation of honeycomb monoliths is shown in Figure S9.

### 3.4. Low-Temperature Nitrogen Adsorption

The textural properties of the monolith samples were investigated by low-temperature nitrogen adsorption using a surface area and porosimetry analyzer (Autosorb, Boynton Beach, FL, USA).

Prior to analysis, the samples were degassed at 150 °C for 3 h under vacuum at a residual pressure below 0.001 mmHg. Nitrogen adsorption–desorption isotherms were recorded at 77 K over a relative pressure range of 0.005–0.995.

Upon preliminary outgassing of the samples at 150 °C for 3 h and residual pressure lesser than 0.001 mmHg, the nitrogen adsorption–desorption isotherms were recorded at the temperature of liquid nitrogen (77 °K) within the range of relative pressures from 0.005 to 0.995.

The specific surface area was determined using the Brunauer–Emmett–Teller (BET) method. The pore size distribution in the range of 1.7–300 nm was calculated from the adsorption branch of the isotherm using the Barrett–Joyner–Halenda (BJH) method. The quenched solid density functional theory (QSDFT) method, assuming a slit/cylindrical equilibrium pore model, was used to determine the total pore volume (V_QSDFT_), surface area (S_QSDFT_), and pore size distribution.

### 3.5. Mercury Intrusion Porosimetry

Mercury intrusion porosimetry (MIP) was used to characterize the pore structure of the carbon monoliths. Prior to analysis, the samples were degassed overnight in a vacuum oven at 150 °C. Mercury intrusion measurements were performed using a Poremasterporosimeter (Quantachrome Instruments, Hook, UK). The pore size distribution, pore volume, bulk density, and surface area were calculated from the intrusion data using the Washburn equation, assuming cylindrical pores. The data were processed using Quantachrome Poremaster data analysis software (Version 8.01). Mesopore volume was determined for pores with diameters below 50 nm.

### 3.6. Scanning Electron Microscopy (SEM) and Energy-Dispersive X-Ray Spectroscopy (EDS)

The morphology and elemental composition of the carbon monoliths were examined by scanning electron microscopy (SEM) coupled with energy-dispersive X-ray spectroscopy (EDS). Prior to analysis, the monoliths were cut into longitudinal sections to expose the internal carbon structure, mounted on aluminum stubs, and coated with a 4 nm platinum layer using a Quorum Q150TES sputter coater (Laughton, East Sussex, UK). SEM images and EDS spectra were acquired using a Zeiss Sigma field-emission scanning electron microscope (Zeiss NTS, Oberkochen, Germany) equipped with an Oxford Instruments EDS system incorporating an X-Max 80 mm^2^ silicon drift detector (SDD). SEM imaging was performed at an accelerating voltage of 5 kV, while the EDS spectra were acquired and processed using Aztec software v5.0. Semi-quantitative elemental composition was determined from the EDS spectra.

### 3.7. CHNS Elemental Analysis

Carbon, hydrogen, nitrogen, and sulfur contents were determined using a VARIO MICRO CUBE CHNS elemental analyzer (Elementar, Hanau, Germany) according to the manufacturer’s standard operating procedure.

### 3.8. FTIR Spectroscopy

The surface functional groups of the carbon adsorbents were analyzed by Fourier transform infrared (FTIR) spectroscopy. Approximately 10 mg of each sample was finely ground with 200 mg of spectroscopic-grade KBr using an agate mortar and pestle until a homogeneous mixture was obtained. The mixture was pressed into transparent pellets and analyzed using a PerkinElmer Spectrum 65 FTIR spectrometer (Shelton, CT, USA) over the wavenumber range of 4000–450 cm^−1^.

### 3.9. Analysis of the Cytotoxicity of Monoliths

The cytotoxicity of monoliths was evaluated by using their extracts in MTS and LDH assays for cell viability/lysis adapted in-house for assessing materials with large surface area and adsorption capacity as described in [35]. The MTS (tetrazolium compound) assay (Cell Titer 96 Aqueous One Solution Cell Proliferation Assay solution Promega) is a colorimetric method for assessing cell viability, as the cellular oxidoreductase enzyme can reduce the tetrazolium compound to a soluble formazan product which can reflect the number of viable cells present. On the other hand, the lactase dehydrogenase (LDH) assay (CytoTox 96^®^ Non-Radioactive Cytotoxicity Assay, Promega, Madison, WI, USA) detects the stable LDH enzyme common in all cells and is measured when cell membranes are no longer intact (cytolysis). The extracts were prepared by placing materials (0.1 g) into media (1 mL) with or without fetal calf serum (FCS) at 37 °C (5% CO_2_) for 24 h, with 50–100% extract solutions measured to circumvent the large surface area and ability of the monolithic sorbents to adsorb essential nutrients from the media and thus influence cell growth, giving false positive results. The extracts were filtered using a 0.45 μm pre-conditioned surfactant-free cellulose acetate syringe filter to sterilize the samples and 100 μL aliquots (in triplicate) were incubated at 37 °C (5% CO_2_) for 24 h with *V79* cells (Chinese hamster lung, male, ECACC No. 86041102) pre-seeded into 96-well plates. Experiments were carried out using duplicate technical repeats and triplicate experimental repeats. Results were compared to a negative control (no material extract) and a positive control of a PVC polymer containing 0.57% Dibutyltin maleate (HydroPolymers Ltd., Durham Co., Newton Aycliffe, UK).

### 3.10. Hemocompatibility Testing of Monoliths

#### 3.10.1. Experimental System for Continuous Blood Circulation Through Monolith

Small prototype monoliths-C were connected to a peristaltic pump via silicone tubing and 0.9% sterile NaCl solution was passed through the monoliths at a rate of 2.5 mL/min for 2 h to pre-equilibrate the system prior to the blood circulation. Healthy volunteer blood was collected by two separate procedures in accordance with the requirement of the individual hemocompatibility tests. Whole blood was collected into either sodium citrate (3.2%) 9 mL Vacutainer^®^ blood collection tubes (BD Biosciences, Wokingham, Berkshire, UK) after the initial 2 mL was discarded (activated platelets), or into EDTA (1.5 mg/L) 9 mL Vacutainer^®^ tubes. A volume of 20 mL of blood was then circulated through two monoliths and a silicone tubing-only control at a rate of 5 mL/min, with samples (2 mL) collected at time 0, after one passage through the monolith and then after 30 and 60 min of continuous circulation through the monolith. The flow rate chosen was to simulate hemodialysis, with an average of 15 passages of blood through the monolith by the 60 min time point in comparison to ~12 for a typical 3 h hemodialysis session. The experiment was repeated 3 times for each anti-coagulant with different donors and the results were pooled (*n* = 5 monoliths total).

The ethics approval for these experiments was obtained from the Ethics Committee of the University of Brighton. Healthy volunteers (for blood samples) were fully informed of the purposes of the research, ensured that their participation was voluntary, and gave their consent to take part on the condition of anonymity, confidentiality, and security of data storage.

#### 3.10.2. Platelet Activation Assay

An important inflammatory parameter to measure when determining the hemocompatibility of a material is that of platelet activation. The platelet activation assay measures the activated platelet population in the whole platelet population using a three-color stain single analysis and flow cytometry. The three surface markers measured included: membrane glycoprotein GpIIIa (CD61) to identify platelets, and anti-activated glycoprotein 11b/11a fibrinogen binding site marker (PAC1) and p-selectin (CD 62-P) to identify activated platelets. Citrate blood samples of 5 μL were incubated with 7.5 μL of each of the three fluorescent antibodies, PE *Mouse Anti-Human CD61*, APC *Mouse Anti-Human CD62P*, and FITC *Mouse Anti-Human PAC-1* (BD Biosciences, Wokingham, Berkshire, UK), for 20 min in the dark. Samples were then fixed with a chilled 1% paraformaldehyde solution in phosphate-buffered saline (PBS) for 30 min before analysis using the Accuri C6 flow cytometer (Beckton Dickinson, Franklin Lakes, NJ, USA). A positive control of blood activated by phorbol myristate acetate (PMA, final concentration 100 nM) and negative control at time zero were prepared and stained using the identical procedure.

#### 3.10.3. Granulocyte and Monocyte Activation Assay

The granulocyte and monocyte activation assay measures both granulocytes and monocytes in the whole blood population using a two-color stain single analysis. The cells express on the surface of the CD14-linked membrane glycoprotein, also known as the LPS receptor that is expressed at higher levels on monocytes than granulocytes, and the marker CD11b, which is expressed on activated granulocytes and monocytes. Citrate blood samples of 100 μL were incubated with 10 μL of the two fluorescent antibodies, APC *Mouse Anti-Human CD11b/Mac-1* and PE *Mouse Anti-Human CD14* (BD Biosciences, Wokingham, Berkshire, UK), in the dark for 20 min. Samples were then incubated with a chilled Lyse/Fix Buffer (BD Biosciences, Wokingham, Berkshire, UK) for 30 min before centrifugation at 500× *g* for 6 min, and the pellet was re-suspended in 300 μL of stain buffer (2% fetal bovine serum in PBS) before analysis using the Accuri C6 flow cytometer (Beckton Dickinson. Franklin Lakes, NJ, USA). A positive control of blood activated with recombinant human IL-6 and IL-8 (BD Biosciences, final concentration 100 ng/mL) and negative control at time zero were prepared and stained using identical procedures.

#### 3.10.4. Coagulation Assay

The standard coagulation tests for hemocompatibility: Partial thromboplastin time (PTT), to measure intrinsic and common clotting pathways, prothrombin time (PT), to measure extrinsic and common clotting pathway activation, Thrombin time (TT), to measure the final step of the common coagulation pathway, and the fibrinogen concentration were used to evaluate the activation of the intrinsic and extrinsic coagulation pathways during blood circulation through the monolith, which could lead to the formation of fibrin clots [60]. Volumes of 1 mL of citrate blood samples were centrifuged at 2500× *g* for 15 min and the plasma was stored at −80 °C before analysis. The PTT Automate kit (PTT test), Neoplastine CI plus kit (PT test), STA Thrombin kit (TT test), and Fibri-prest (fibrinogen concentration test) (Diagnostica Stago, Theale, Berkshire, UK) were carried out following manufacturer’s instructions in addition to the unicalibrator and Coag N and P assay controls that accompany the kits. Coagulation times were measured using the STart^®^ Hemostasis benchtop Analyzer (Diagnostica Stago, Theale, Berkshire, UK).

#### 3.10.5. Complement Activation Assay

The complement activation assay was used to assess the hemocompatibility of the monolith by measuring the complement components C3a, C4a and C5a, which are released during complement activation and then rapidly converted into the more stable C3a-des-Arg, C4ades-Arg and C5a-des-Arg forms. Blood samples of 1 mL with EDTA were collected and 10 μL of FUT-175 (5 mg/mL, Sigma, Gillingham, Dorset, UK) was added to inhibit the continual ex vivo complement activation, reflecting complement levels at the time of collection [61]. Samples were centrifuged at 2500× *g* for 15 min and the plasma was removed and stored at −80 °C before use. The concentration of the complement components C3a-des-Arg, C4a-des-Arg, and C5a-des-Arg were measured with the CBA Human anaphylatoxin kit (BD Biosciences, Wokingham, Berkshire, UK) following manufacturer’s instructions and measured using the Accuri C6flow cytometer (BD Biosciences, Franklin Lakes, NJ, USA). A positive control of blood activated with Zymosan A (33 μg/mL final concentration, Sigma, Gillingham, Dorset, UK) and negative control at time zero were prepared and stained using identical procedures.

#### 3.10.6. Hemolysis Assay

Hemolysis of red blood cells can be caused by the release of toxic substances from the surface of a biomaterial, or the activation of platelets and leukocytes resulting in the release of factors causing membrane disruption and hemoglobin (Hg) release. A volume of 1 mL of citrate blood was centrifuged at 2500× *g* for 15 min and the plasma was removed and stored at −80 °C before use. Plasma samples were tested using a standard hemolysis kit (Sigma, Gillingham, Dorset, UK) and the concentration of Hg was calculated in mg/dL.

### 3.11. Assessment of PBUTs and Inflammatory Cytokines Elimination by Carbon Monoliths in a Recirculation Flowing System

The adsorption characteristics of the RH-derived nanoporous carbon monoliths were assessed for their adsorptive capacity for a range of biological toxins (PBUTs and inflammatory cytokines) in a recirculation system using fresh frozen human plasma to mimic the process of hemoperfusion in the clinic.

Carbon monoliths-C, monoliths-N and monoliths-P (mean *n* = 3 for each experiment, i.e., in triplicates) were connected via silicon tubing from a reservoir through a peristaltic pump set at a flow rate of 5 mls/min and pre-equilibrated with 20 mL of phosphate-buffered saline (PBS) for 2 h. The PBS was then removed from the reservoir and tubing. Fresh frozen human plasma supplemented with PCS (1 mM), IS (0.5 mM), IL-6 (1000 pg/mL), and IL-8 (1000 pg/mL) in each separate single-component adsorption experiment was incubated in a shaking incubator at 37 °C for 1 h. After the incubation 20 mL of spiked plasma was added to each reservoir for the monoliths and controls (tubing only), the peristaltic pump was set at a flow rate of 5 mls/min. At the 5, 30, 60, 90 and 120 min time points, 1 mL of plasma was collected from each and separated into aliquots for ELISA and HPLC analysis and stored in a −80 °C freezer before analysis. An enzyme-linked immunoadsorbent assay (ELISA) was used to determine the concentration of the inflammatory cytokines IL-6 and IL-8 in the plasma samples according to manufacturer’s instructions (BD Biosciences, Wokingham, Berkshire, UK). An HPLC technique was used to determine the concentration of the uremic toxins in the plasma samples.

All experiments were performed in triplicate, and the results were presented as the average. A uremic toxin removal efficiency (RE, %) of an adsorbate was calculated according to Equation (2), while the amounts of endotoxin adsorbed per mass unit or adsorption capacity, qt, were computed using Equation (3) (Figure S3), respectively [17]:

(2) $$RE(\%)=\frac{\left(C_{0}-C_{t}\right)}{C_{0}}\times 100$$

(3) $$q_{t}(mg/g)=\frac{\left(C_{0}-C_{t}\right)\times V}{W}$$

where C_0_ and C_t_ (mM/L) for PCS and IS, and (pg/mL) for IL-6 and IL-8 are the initial and final concentrations at respective time points (t, min) of an adsorbate solution. *V* is the volume of the human plasma solution (20 mL), and *W* (g) is an average weight of monoliths, e.g., 1.13 g for monolith-C, 1.09 g for monolith-N, and 1.34 g for monolith-P.

#### 3.11.1. HPLC Analysis of PCS and IS Removal by Carbons

An HPLC method was adapted from that described by de Loor et al. [62] using competitive albumin binding with sodium octanoate to measure total PCS and IS in plasma. A volume of 40 µL of each sample was added to 160 µL of plasma (1:5 dilution) before incubation with 250 µL of sodium ocatanoate (0.24 µM) for 30 min. A volume of 20 µL of internal standard naphthalene sulfonic acid (0.5 mM) was added. Samples were mixed with 2 mL of ice-cold acetone to precipitate proteins and were centrifuged for 10 min at 4000 rpm. The supernatant was removed, mixed with 2 mL ice cold dichloromethane, and again centrifuged for 10 min at 4000 rpm. The top aqueous phase was removed and 40 µL of 1 M HCl was added to stabilize the solution. HPLC analysis of samples was carried out using a Fortis column, Waters 717 plus Autosampler set at 15 °C, a Perkin Elmer Series 200 oven and LC pump set at 25 °C, a Varian Prostar model 360 fluorescent detector, and a PE Nelson 900 Series interface. A 10 µL loading volume was used. The mobile phase of acetonitrile mixed with TFA (0.2%) and water mixed with TFA (0.2%) was run on a gradient program at a variable flow rate from 0.6 to 1 mL/minute. Dual detection wavelengths were used. IS and internal standard were detected first at an excitation/emission wavelength setting of 280 nm/360 nm. Detection of PCS was carried out at an excitation/emission wavelength setting of 260 nm/296 nm. Standard curves of PCS and IS were constructed with a concentration range from 0 to 250 mM and 0 to 125 mM respectively (Figure S9). Upon proper dilution, these standard curves were then used to calculate the concentrations of PCS and IS in spiked plasma samples incubated with different carbon monoliths over time.

#### 3.11.2. ELISA Analysis of Inflammatory Cytokine Removal by Monoliths

An enzyme-linked immunoadsorbent assay (ELISA) was used to determine the concentration of the inflammatory cytokines, IL-6 and IL-8, in the plasma samples. Samples were diluted in assay diluent prior to analysis by the IL-6 and IL-8 ELISA set (BD Biosciences, Wokingham, Berkshire, UK). The ELISAs were conducted following the manufacturer’s instructions. Briefly, the 96-well ELISA plates were coated with capture antibody overnight before a washing step and addition of a blocking agent using diluted fetal bovine serum (10%). After a second washing stage, the standards and diluted samples were added to the plate and incubated for 2 h, before another washing step and addition of the secondary antibody and enzyme reagent. After further incubation, the TMB substrate reagent was added and the plate was allowed to develop, before the stop reagent was added and the plate was measured on a microplate reader set at an absorbance of 450 nm. Upon proper dilution, the unknown concentrations of the samples were then calculated using the standard curves (Figure S10).

## 4. Conclusions

The study suggests that the porosity data and SEM images of the CO_2_-activated and successive desilicated monolith-C show that the monolith has a suitable porous structure to act as the adsorbent in a hemoperfusion device. The standard assessment of platelet activation, coagulation, complement activation, granulocyte/monocyte activation, and hemolysis following healthy donor blood filtration through the monolith-C demonstrates its good hemocompatibility. The monolith-C does not activate the inflammatory response or cause blood coagulation or hemolysis to any further degree.

Surface ammoxidation of the monolith-C, to produce the monolith-N, resulted in only a minor decrease in BET-specific surface area (816 to 790 m^2^/g) and micropore volume (0.407 to 0.381 cm^3^/g), indicating preservation of the carbon framework. Hence, BET-specific surface area of the monolith-N decreases by 5–10%; the total QSDFT pore volume decreases from 0.617 cm^3^/g to 0.542 cm^3^/g. Alternatively, the monolith-P was produced from the phosphorylated RH powder at 450 C^0^, followed by mixing with lignin, extrusion, and successive carbonization; its BET-specific surface area is 832 m^2^/g, its micropore volume is 0.447 cm^3^/g, and its QSDFT pore volume is 0.845 cm^3^/g. The cytotoxicity studies using the modified MTS and LDH methods with extracts from three types of monoliths, monolith-C, monolith-N and monolith-P, demonstrated that they did not cause any changes to the cell viability or cell lysis and thus could be considered non-cytotoxic. Nevertheless, removal efficiency for these adsorbates do not correlate with the LTNA and MIP study results. Hence, it was found that monoliths are able to eliminate biologically relevant PBUTs and inflammatory cytokines levels; e.g., adsorption removal within 5 to 120 min of circulation reached the values of: PCS—12.22–30.14% (monolith-C), 10.15–40.05% (monolith-N), 7.87–35.11% (monolith-P); IS—17.32–45.24% (monolith-C), 9.22–50.07% (monolith-N), 8.42–55.2% (monolith-P); IL-6—4.5–10.3% (monolith-C), 7.5–28.4% (monolith-N), 8.3–19.2% (monolith-P); and IL-8—4.8–26.4% (monolith-C), 5.1–37.4% (monolith-N), 8.3–54.5% (monolith-P). This phenomenon for IL-6 and IL-8 falls within the difference of their pI values, i.e., negatively charged IL-6 (pI ≈ 5.98) has a higher affinity to positively charged surface amino groups of the monolith-N, while the positively charged IL-8 (pI ≈ 8.11) has a higher affinity to the negatively charged surface phosphate groups of the monolith-P at the physiological pH of human plasma (pI ≈ 7.4). The results presented in this work suggest that the rice-husk-derived activated carbon monolith is a promising material for hemoperfusion applications, suggesting the interplay of physisorption vs. chemisorption that plays key roles in the adsorption mechanism.

## Figures and Tables

**Table 1:** Specific surface area (S) and pore volume (V) of carbon monoliths calculated using different methods *.

Sample Code	S_BET_, m^2^/g	S_QSDFT_, m^2^/g	V_QSDFT_, cm^3^/g	D_QSDFT_, nm	V_DR_, cm^3^/g	D_DR_, nm	V_BJH_, cm^3^/g	V_MIP≤50nm_ cm^3^/g	V_MIP≤100nm_ cm^3^/g
Monolith-C	816	1101	0.617	0.57	0.407	1.2	0.382	0.136	0.206
Monolith-N	790	1040	0.542	0.55	0.381	1.2	0.292	0.05	0.086
CRH-P-450	1700	1400	1.56	0.82	0.48	---	1.15	---	---
Monolith-P	832	794	0.845	0.57	0.447	1.7	0.65	0.16	0.24

* BET—Brunauer–Emmett–Teller (BET) theory; DFT—quenched solid density functional theory; BJH—Barrett–Joyner–Halenda method; DR—Dubinin–Radushkevich equation; MIP—mercury intrusion porosimetry analysis.

**Table 2:** Coagulation test results for monolith and tubing circulation samples and negative control at time zero measured by the APPT, PT and Thrombin generation times and fibrinogen concentration in one out of three separate experiments with different donor blood plasma (mean *n* = 2, monoliths).

Sample	APPT (s)	PT (s)	Thrombin (s)	Fibrinogen (g/L)
Negative control time = 0	35.80	11.95	15.75	3.72
Monolith single pass	37.78	11.98	15.43	3.50
Monolith −30 min	36.70	11.83	15.13	3.71
Monolith −60 min	36.45	11.90	14.78	3.31
Tubing single pass	37.40	11.95	16.80	3.77
Tubing −30 min	38.35	11.90	16.05	3.25
Tubing −60 min	38.75	12.15	15.95	3.17

**Table 3:** Textural properties and adsorption removal rates of uremic toxins by the monoliths.

Monolith Code	Nitrogen Adsorption and Mercury Porosimetry Data				PBUTs RE/q_120min_, %/mg × g^−1^		Interleukins RE/q_120min_, %/ng × g^−1^	
	S_DFT_, m^2^/g	V_DFT_, cm^3^/g	V_BJH_, cm^3^/g	V_MIP_, cm^3^/g <100 nm	PCS	IS	IL-6	IL-8
C	816	0.617	0.334	0.136	30.14/1.004	45.24	10.3/1.823	26.4/4.673
N	790	0.542	0.286	0.086	40.05/1.383	50.07	28.4/5.211	37.3/6.844
P	832	0.845	0.636	0.241	35.11/0.986	55.2	19.2/2.866	54.4/8.134

**Table 4:** Principal chemical features of human IL-6.

Chemical Motif	Representative Residues	Characteristic Chemical Properties
Negatively charged residues	Asp, Glu	Electrostatic interactions (negative charge)
Positively charged residues	Lys, Arg, His	Electrostatic interactions (positive charge)
Aromatic residues	Phe, Tyr, Trp, His	π–π and hydrophobic interactions
Polar hydroxyl residues	Ser, Thr, Tyr	Hydrogen bond donor/acceptor
Amide-containing residues	Asn, Gln	Hydrogen bond donor/acceptor
Sulfur-containing residues	Cys, Met	Disulfide bond formation/hydrophobic interactions
Aliphatic hydrophobic residues	Ala, Val, Leu, Ile, Met	Hydrophobic interactions

**Table 5:** Principal chemical features of human IL-8.

Chemical Motif	Representative Residues	Characteristic Chemical Properties
Negatively charged residues	Asp, Glu	Electrostatic interactions (negative charge)
Positively charged residues	Lys, Arg, His	Electrostatic interactions (positive charge)
Aromatic residues	Phe, Tyr, Trp, His	π–π and hydrophobic interactions
Polar hydroxyl residues	Ser, Thr, Tyr	Hydrogen bond donor/acceptor
Amide-containing residues	Asn, Gln	Hydrogen bond donor/acceptor
Sulfur-containing residues	Cys, Met	Disulfide bond formation/hydrophobic interactions
Aliphatic hydrophobic residues	Ala, Val, Leu, Ile, Met	Hydrophobic interactions

## Data Availability

The original contributions presented in this study are included in the article/Supplementary Materials. Further inquiries can be directed to the corresponding authors.

## Supplementary Materials

The following supporting information can be downloaded at: https://www.mdpi.com/article/10.3390/ijms27177972/s1.

## References

[1] Mutig K., Singh P.B., Lebedeva S. (2026). Interleukin-6 in Natural and Pathophysiological Kidney Aging. Cells.

[2] Vanholder R., Pletinck A., Schepers E., Glorieux G. (2018). Biochemical and Clinical Impact of Organic Uremic Retention Solutes: A Comprehensive Update. Toxins.

[3] Lu C.-L., Zheng C.-M., Lu K.-C., Liao M.-T., Wu K.-L., Ma M.-C. (2021). Indoxyl-Sulfate-Induced Redox Imbalance in Chronic Kidney Disease. Antioxidants.

[4] Vaziri N.D., Zhao Y.-Y., Pahl M.V. (2016). Altered intestinal microbial flora and impaired epithelial barrier structure and function in CKD: The nature, mechanisms, consequences and potential treatment. Nephrol. Dial. Transplant..

[5] Lisowska-Myjak B., Zborowska H., Jaźwiec R., Karlińska M., Skarżyńska E. (2021). Serum indoxyl sulphate and its relation to albumin and α1-acid glycoprotein as a potential biomarkers of maternal intestinal metabolism during pregnancy and postpartum. PLoS ONE.

[6] Rosner M.H., Reis T., Husain-Syed F., Vanholder R., Hutchison C., Stenvinkel P., Blankestijn P.J., Cozzolino M., Juillard L., Kashani K. (2021). Classification of Uremic Toxins and Their Role in Kidney Failure. Clin. J. Am. Soc. Nephrol..

[7] Brydges C.R., Fiehn O., Mayberg H.S., Schreiber H., Dehkordi S.M., Bhattacharyya S., Cha J., Choi K.S., Craighead W.E., Krishnan R.R. (2021). Indoxyl sulfate, a gut microbiome-derived uremic toxin, is associated with psychic anxiety and its functional magnetic resonance imaging-based neurologic signature. Sci. Rep..

[8] Cozzolino M., Magagnoli L., Ciceri P. (2025). From Physicochemical Classification to Multidimensional Insights: A Comprehensive Review of Uremic Toxin Research. Toxins.

[9] Pavlenko D., Giasafaki D., Charalambopoulou G., van Geffen E., Gerritsen K.G.F., Steriotis T., Stamatialis D. (2017). Carbon Adsorbents with Dual Porosity for Efficient Removal of Uremic Toxins and Cytokines from Human Plasma. Sci. Rep..

[10] Sánchez-Ospina D., Mas-Fontao S., Gracia-Iguacel C., Avello A., de Rivera M., Mujika-Marticorena M., Gonzalez-Parra E. (2024). Displacing the Burden: A Review of Protein-Bound Uremic Toxin Clearance Strategies in Chronic Kidney Disease. J. Clin. Med..

[11] Bardi G. (2022). Chemokines and nanomaterials: Interaction for useful immune-applications. Explor. Immunol..

[12] Chermiti R., Bataille S., Giaime P., Solignac J., Pedinielli N., McKay N., Bigey-Frau D., Lano G., Benjelloun H., Addi T. (2025). From Uremic Toxins to Hemodialysis Access Failure: IL-8 and MCP-1 Chemokines as a Link Between Endothelial Activation and AV Access Complications. Toxins.

[13] Zhang J., Yuan Y., An X., Ouyang C., Ren H., Yang G., Yu X., Lv X., Zhang B., Wang N. (2016). Comparison of combined blood purification techniques in treatment of dialysis patients with uraemic pruritus. Int. J. Clin. Exp. Med..

[14] Zheng Y., Pescatore N., Gogotsi Y., Dyatkin B., Ingavle G., Mochalin V., Ozulumba T., Mikhalovsky S., Sandeman S. (2018). Rapid Adsorption of Proinflammatory Cytokines by Graphene Nanoplatelets and Their Composites for Extracorporeal Detoxification. J. Nanomater..

[15] Magnani S., Atti M. (2021). Uremic Toxins and Blood Purification: A Review of Current Evidence and Future Perspectives. Toxins.

[16] Botella J., Ghezzi P.M., Sanz-Moreno C. (2000). Adsorption in hemodialysis. Kidney Int..

[17] Sandeman S.R., Zheng Y., Ingavle G.C., Howell C.A., Mikhalovsky S.V., Basnayake K., Boyd O., Davenport A., Beaton N., Davies N. (2017). A haemocompatible and scalable nanoporous adsorbent monolith synthesised using a novel lignin binder route to augment the adsorption of poorly removed uraemic toxins in haemodialysis. Biomed. Mater..

[18] Jandosov J., Berillo D., Misra A., Alavijeh M., Chenchik D., Baimenov A., Bernardo M., Azat S., Mansurov Z., Silvestre-Albero J. (2025). Biomass-Derived Nanoporous Carbon Honeycomb Monoliths for Environmental Lipopolysaccharide Adsorption from Aqueous Media. Int. J. Mol. Sci..

[19] Jandosov J.M., Mansurov Z.A., Biisenbayev M.A., Ismagilov Z.R., Shikina N.V., Ismagilov I.Z., Konuspayeva S.R., Shaymardan M. (2012). Mesoporous Carbon-Based Rhodium Catalysts for Benzene Hydrogenation. Eurasian Chem.-Technol. J..

[20] Jandosov J., Shikina N., Bijsenbayev M., Shamalov M., Ismagilov Z., Mansurov Z. (2009). Evaluation of Synthetic Conditions for H3PO4 Chemically Activated Rice Husk and Preparation of Honeycomb Monoliths. Eurasian Chem.-Technol. J..

[21] Merkel A., Satayeva A., Cannon F., Howell C., Meikle S., László K., Inglezakis V., Jandosov J., Ray S., Mansurov Z. (2016). Characterisation of activated carbons obtained from rice husk. Eurasian Chem.-Technol. J..

[22] Dias D., Don D., Jandosov J., Bernardo M., Pinto F., Fonseca I., Sanches A., Caetano P.S., Lyubchyk S., Lapa N. (2021). Highly efficient porous carbons for the removal of W(VI) oxyanion from wastewaters. J. Hazard. Mater..

[23] Jandosov J., Mansurov Z., Biisenbayev M., Kerimkulova A., Ismagilov Z., Shikina N., Ismagilov I., Andrievskaya I. (2011). Mesoporous Composite Materials from Acivated Rice Husk Carbon and Montmorillonite. Eurasian Chem.-Technol. J..

[24] Jandosov J., Mikhalovska L., Howell C., Chenchik D., Kosher B., Lyubchik S., Silvestre-Albero J., Ablaikhanova N., Srailova G., Tuleukhanov S. (2017). Synthesis, Morphostructure, Surface Chemistry and Preclinical Studies of Nanoporous Rice Husk-Derived Biochars for Gastrointestinal Detoxification. Eurasian Chem.-Technol. J..

[25] Jandosov J., Mansurov Z.A., Bijsenbayev M.A., Tulepov M.I., Ismagilov Z.R., Shikina N.V., Ismagilov I.Z., Andrievskaya I.P. (2013). Synthesis of Microporous-Mesoporous Carbons from Rice Husk via H_3_PO_4_-Activation. Adv. Mater. Res..

[26] Mozaffari A., Alimohammadi F., Gashti M.P. (2025). Functional Carbon-Based Materials for Blood Purification: Recent Advances Toward Improved Treatment of Renal Failure and Patient Quality of Life. Bioengineering.

[27] Wu S., Yue P., Ma Y., Zou Y., Liang W., Ye Q. (2025). Hemoperfusion Adsorbents for Removal of Common Toxins in Liver and Kidney Failure: Recent Progress, Challenges, and Prospects. Adv. Mater..

[28] (2009). Biological Evaluation of Medical Devices–Part 5: Tests for In Vitro Cytotoxicity.

[29] (2017). Biological Evaluation of Medical Devices Part 4: Selection of Tests for Interactions with Blood.

[30] Seyfert U.T., Biehl V., Schenk J. (2002). In vitro hemocompatibility testing of biomaterials according to the ISO 10993-4. Biomol. Eng..

[31] Thommes M., Kaneko K., Neimark A.V., Olivier J.P., Rodriguez-Reinoso F., Rouquerol J., Sing K.S.W. (2015). Physisorption of gases, with special reference to the evaluation of surface area and pore size distribution (IUPAC Technical Report). Pure Appl. Chem..

[32] Jandosov J., Chenchik D., Baimenov A., Silvestre-Albero J., Bernardo M., Azat S., Doszhanov Y., Sabitov A., Busquets R., Howell C. (2026). Assessment of Phenolic and Indolic Compounds Removal from Aqueous Media Using Lignocellulose-Derived Surface-Modified Nanoporous Carbon Adsorbents: A Comparative Study. Int. J. Mol. Sci..

[33] Bang G.S., Shim G.W., Shin G.H., Jung D.Y., Park H., Hong W.G., Choi J., Lee J., Choi S.-Y. (2018). Pyridinic-N-Doped Graphene Paper from Perforated Graphene Oxide for Efficient Oxygen Reduction. ACS Omega.

[34] Puziy A.M., Poddubnaya O.I., Gawdzik B., Tascón J.M.D. (2020). Phosphorus-containing carbons: Preparation, properties and utilization. Carbon.

[35] Barnes L.-M., Phillips G., Davies J., Lloyd A., Cheek E., Tennison S.R., Rawlinson A., Kozynchenko O., Mikhalovsky S. (2009). The cytotoxicity of highly porous medical carbon adsorbents. Carbon.

[36] Wolf M.F., Coleman K.P., Rankin E.A., Lewerenz G.M., Wagner W.R., Sakiyama-Elbert S.E., Zhang G., Yaszemski M.J.B.T.-B.S. (2020). 2.3.3—In Vitro Assessment of Cell and Tissue Compatibility. Biomaterials Science.

[37] Nonckreman C.J., Fleith S., Rouxhet P.G., Dupont-Gillain C.C. (2010). Competitive adsorption of fibrinogen and albumin and blood platelet adhesion on surfaces modified with nanoparticles and/or PEO. Colloids Surf. B Biointerfaces.

[38] Sandeman S.R., Howell C.A., Phillips G.J., Lloyd A.W., Davies J.G., Mikhalovsky S.V., Tennison S.R., Rawlinson A.P., Kozynchenko O.P., Owen H.L.H. (2005). Assessing the in vitro biocompatibility of a novel carbon device for the treatment of sepsis. Biomaterials.

[39] Moen O., Fosse E., Bråten J., Andersson C., Fagerhol M.K., Venge P., Høgåsen K., Mollnes T.E. (1994). Roller and centrifugal pumps compared in vitro with regard to haemolysis, granulocyte and complement activation. Perfusion.

[40] Butler J., Rocker G.M., Westaby S. (1993). Inflammatory response to cardiopulmonary bypass. Ann. Thorac. Surg..

[41] McNally A.K., Anderson J.M. (1994). Complement C3 participation in monocyte adhesion to different surfaces. Proc. Natl. Acad. Sci. USA.

[42] Liu Y., Yang Y., Wu F. (2010). Effects of L-arginine immobilization on the anticoagulant activity and hemolytic property of polyethylene terephthalate films. Appl. Surf. Sci..

[43] Kainthan R., Gnanamani M., Ganguli M., Ghosh T., Brooks D., Maiti S., Kizhakkedathu J. (2006). Blood compatibility of novel water soluble hyperbranched polyglycerol-based multivalent cationic polymers and their interaction with DNA. Biomaterials.

[44] Díaz-Rodríguez P., González P., Serra J., Landin M. (2014). Key parameters in blood-surface interactions of 3D bioinspired ceramic materials. Mater. Sci. Eng. C Mater. Biol. Appl..

[45] Sadrzadeh S.M., Graf E., Panter S.S., Hallaway P.E., Eaton J.W. (1984). Hemoglobin. A biologic fenton reagent. J. Biol. Chem..

[46] Zimmerman J.J. (1999). Deciphering the dark side of free hemoglobin in sepsis. Crit. Care Med..

[47] Morgan I.S., Codispoti M., Sanger K., Mankad P.S. (1998). Superiority of centrifugal pump over roller pump in paediatric cardiac surgery: Prospective randomised trial. Eur. J. Cardio-Thorac. Surg..

[48] Moen O., Fosse E., Dregelid E., Brockmeier V., Andersson C., Høgåsen K., Venge P., Mollnes T.E., Kierulf P. (1996). Centrifugal pump and heparin coating improves cardiopulmonary bypass biocompatibility. Ann. Thorac. Surg..

[49] Khubutiya M.S., Tsivadze A.Y., Garzaeva G.R., Andreev V.N., Goldin M.M. (2013). Adsorption interaction of individual hemoglobin with active carbon surface modified with polypyrrole. Prot. Met. Phys. Chem. Surf..

[50] Pfeifer P.H., Kawahara M.S., Hugli T.E. (1999). Possible mechanism for in vitro complement activation in blood and plasma samples: Futhan/EDTA controls in vitro complement activation. Clin. Chem..

[51] Belmont H.M., Hopkins P., Edelson H.S., Kaplan H.B., Ludewig R., Weissmann G., Abramson S. (1986). Complement activation during systemic lupus erythematosus. C3a and C5a anaphylatoxins circulate during exacerbations of disease. Arthritis Rheum..

[52] Abou-Ragheb H.H., Williams A.J., Brown C.B., Milford-Ward A. (1991). Plasma levels and mode of excretion of the anaphylatoxins C3a and C4a in renal disease. J. Clin. Lab. Immunol..

[53] Cheung A.K., Parker C.J., Wilcox L.A., Janatova J. (1990). Activation of complement by hemodialysis membranes: Polyacrylonitrile binds more C3a than cuprophan. Kidney Int..

[54] Mertowska P., Mertowski S., Smarz-Widelska I., Grywalska E. (2022). Biological Role, Mechanism of Action and the Importance of Interleukins in Kidney Diseases. Int. J. Mol. Sci..

[55] Messina J.M., Luo M., Hossan M.S., Gadelrab H.A., Yang X., John A., Wilmore J.R., Luo J. (2024). Unveiling cytokine charge disparity as a potential mechanism for immune regulation. Cytokine Growth Factor Rev..

[56] Joshi A., Xiao Z., Suman S., Cooper C., Ha K., Carson J.A., Quarles L.D., Smith J.C., Gupta M. (2025). Structure-Based Design of Small-Molecule Inhibitors of Human Interleukin-6. Molecules.

[57] Somers W., Stahl M., Seehra J.S. (1997). 1.9 Å crystal structure of interleukin 6: Implications for a novel mode of receptor dimerization and signaling. EMBO J..

[58] Tran T.-T.-N., Tran Q.-H., Nguyen Q.-T., Le M.-T., Trinh D.-T.T., Thai K.-M. (2022). Identification of potential interleukin-8 inhibitors acting on the interactive site between chemokine and CXCR2 receptor: A computational approach. PLoS ONE.

[59] Ortega-Ortiz H., Bernardo M., Matos I., Fonseca I., Mota J.P.B., Jandosov J., Ribeiro R.P.P.L. (2026). Rice husk-based activated carbon honeycomb monolith for biogas upgrading and CO2 capture from flue gases. Adsorption.

[60] Kung F.-C., Yang M.-C. (2006). Effect of conjugated linoleic acid immobilization on the hemocompatibility of cellulose acetate membrane. Colloids Surf. B Biointerfaces.

[61] Ahmed A.E., Peter J.B. (1995). Clinical utility of complement assessment. Clin. Diagn. Lab. Immunol..

[62] De Loor H., Meijers B.K.I., Meyer T.W., Bammens B., Verbeke K., Dehaen W., Evenepoel P. (2009). Sodium octanoate to reverse indoxyl sulfate and p-cresyl sulfate albumin binding in uremic and normal serum during sample preparation followed by fluorescence liquid chromatography. J. Chromatogr. A.

