# Supplementary material for: Functional Nanostructured Carbon Honeycomb Monoliths for Hemoadsorption: Preliminary Studies on Biocompatibility, Protein-Bound Uremic Toxins and Inflammatory Cytokines Elimination
Source: Int J Mol Sci. 2026 Sep 7;27(17):7972. doi: 10.3390/ijms27177972 (PMC13566498; doi:10.3390/ijms27177972)
Supplement: Supplementary file 1 [file ijms-27-07972-s001.zip › ijms-4473241-supplementary.pdf]

Supplementary Materials

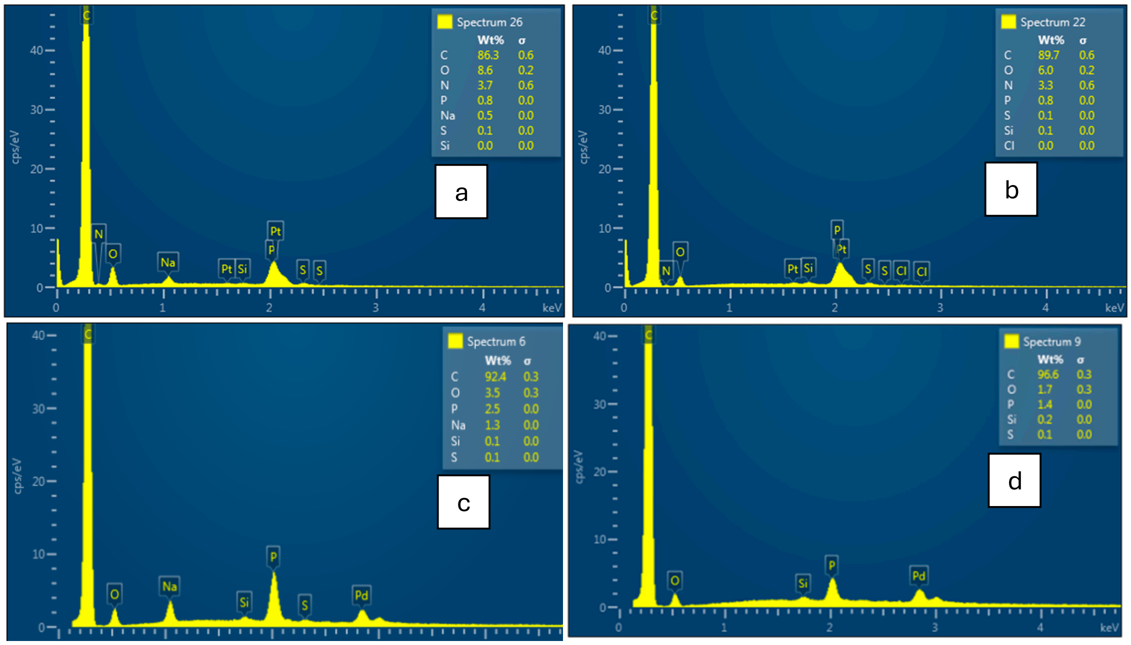

Figure S1. Energy-dispersive X-ray microanalysis of monolith-N (**a**); monolith-N_HCl (**b**); CRH-P-450 (**c**) and monolith-P (**d**).

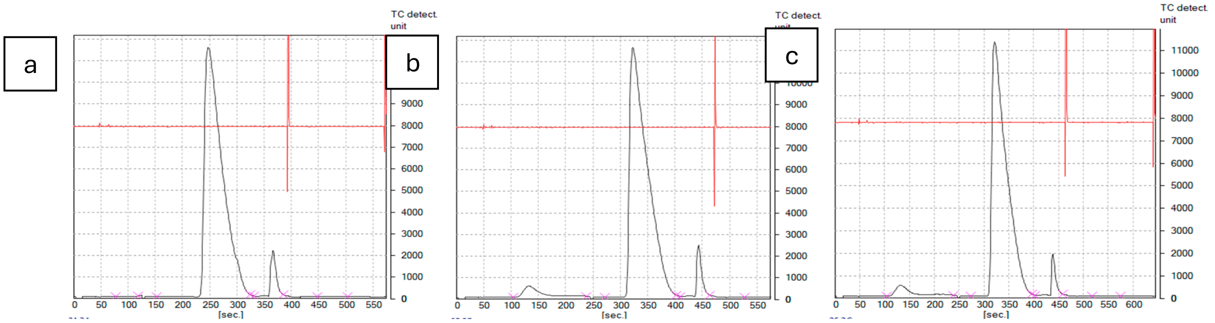

Figure S2. Gas chromatograms, corresponding to the elements C, H, N, S and O (calculated by difference), according to VARIO MICRO CUBE elemental analysis of monoliths. (**a**) monolith-C, (**b**) monolith-N after oxidative ammonolysis, (**c**) monolith-N after treatment with HCl solution.

Table S1. Area of chromatographic peaks corresponding to the content of elements in the monoliths during the modification process, according to elemental CHNOS analysis data.

Monoliths	S_N	S_C	S_H	S_S	N, %	C, %	H, %	O, %	S, %
monolith-C	34	43 792	2 538	32	0.02	92.15	1.89	5.71	0.23
monolith-N	2 136	43 477	2 680	44	3.03	86.7	1.88	8.12	0.27
monolith-N_HCl	1 998	38 463	1 914	30	2.93	79.36	1.44	16.05	0.22

Table S2. Adsorption removal rates of uremic toxins for the monoliths.

		Removal Efficiency, %				
uremic toxins	Time, min	5	30	60	90	120
PCS, mg/g	Monolith-C	12.22	16.32	20.14	26.82	30.14
	Monolith-N	10.15	10.58	29.89	37.19	40.05
	Monolith-P	7.87	14.78	24.33	32.6	35.11
IS, mg/g	Monolith-C	17.32	24.11	35.51	41.32	45.24
	Monolith-N	9.22	10.55	37.87	46.48	50.07
	Monolith-P	8.42	18.06	39.03	50.98	55.2

IL-6, ng/g	Monolith-C	4.5	2.7	8.4	9.6	10.3
	Monolith-N	7.5	13,4	22.4	25.3	28.4
	Monolith-P	8.3	13.1	11.6	16.8	19.2
IL-8, ng/g	Monolith-C	4.8	11.2	15.1	22.5	26.4
	Monolith-N	5.1	13.8	27.9	34.3	37.3
	Monolith-P	8.3	40.4	46.5	52.1	54.5
**Adsorption Capacities for**						
PCS, mg/g	Monolith-C	0.407	0.544	0.671	0.893	1.004
	Monolith-N	0.351	0.365	1.032	1.284	1.383
	Monolith-P	0.221	0.415	0.683	0.916	0.986
IS, mg/g	Monolith-C	0.327	0.455	0.67	0.78	0.854
	Monolith-N	0.181	0.207	0.741	0.909	0.979
	Monolith-P	0.134	0.288	0.621	0.811	0.878
IL-6, ng/g	Monolith-C	0.796	0.478	1.487	1.699	1.823
	Monolith-N	1.376	2.458	4.11	4.642	5.211
	Monolith-P	1.239	1.955	1.731	2.507	2.866
IL-8, ng/g	Monolith-C	0.85	1.982	2.673	3.982	4.673
	Monolith-N	0.936	2.532	5.119	6.294	**6.844**
	Monolith-P	1.239	6.03	6.94	7.776	8.134

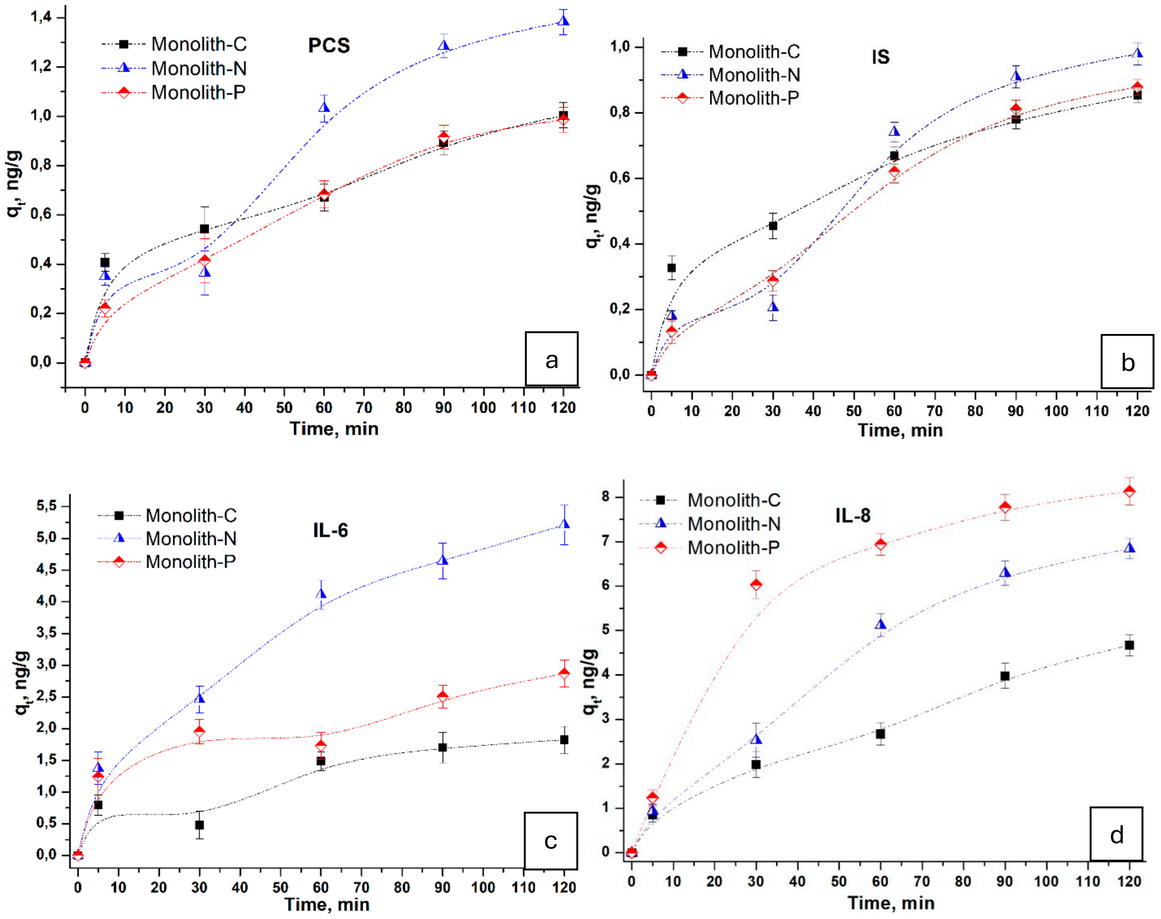

Figure S3. Protein-bound uremic toxins PCS (**a**), IS (**b**) and inflammatory cytokines IL-6 (**c**), IL-8 (**d**) adsorption profiles for the Monolith-C, Monolith-N and Monolith-P over time course of spiked plasma perfusion in recirculating system for 120 min.

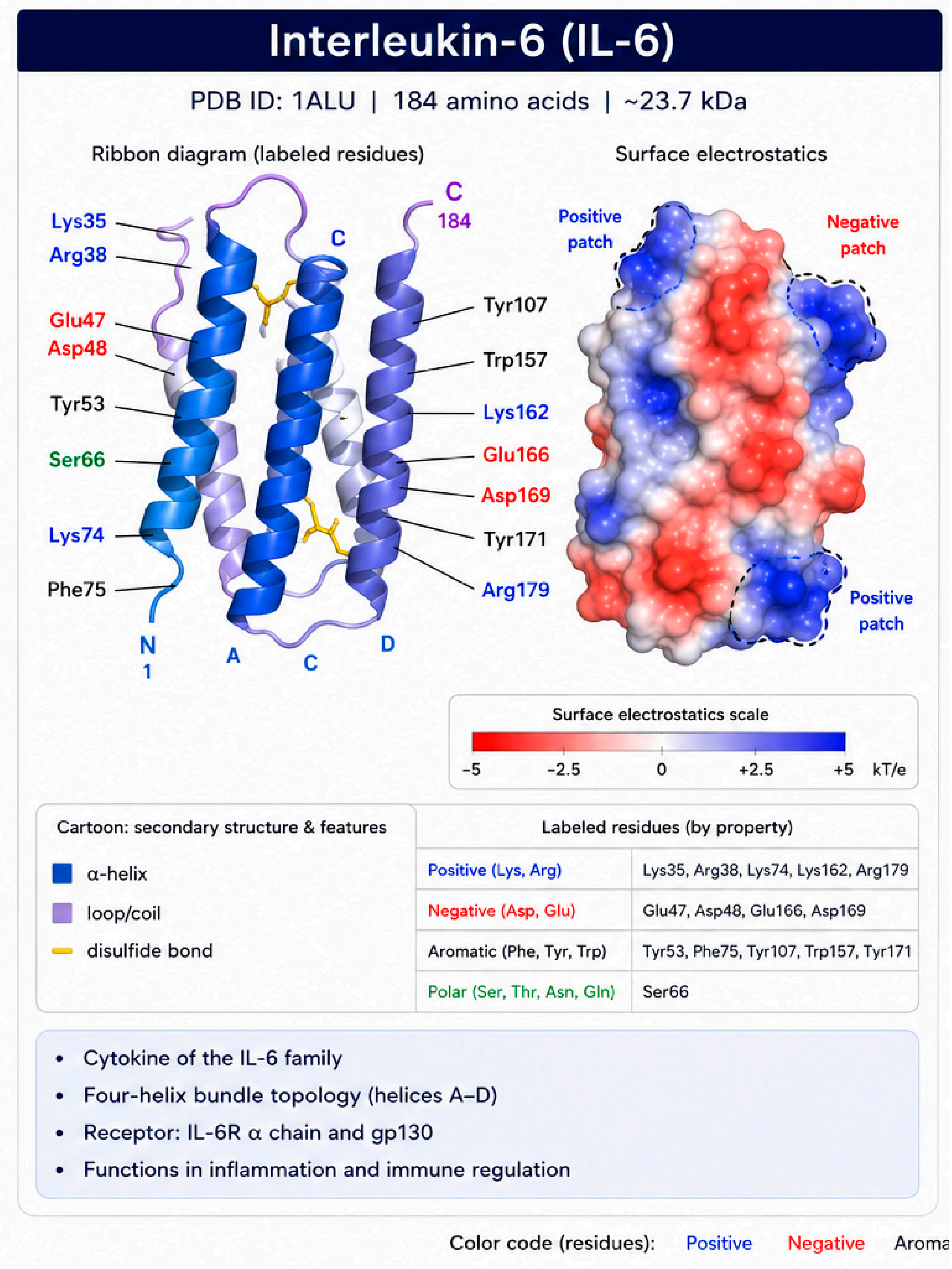

Figure S4. Structural representation of human interleukin-6 (IL-6). Ribbon and electrostatic surface representations with schematic annotation of representative amino acid residues. Created by the authors based on PDB structure 1ALU.

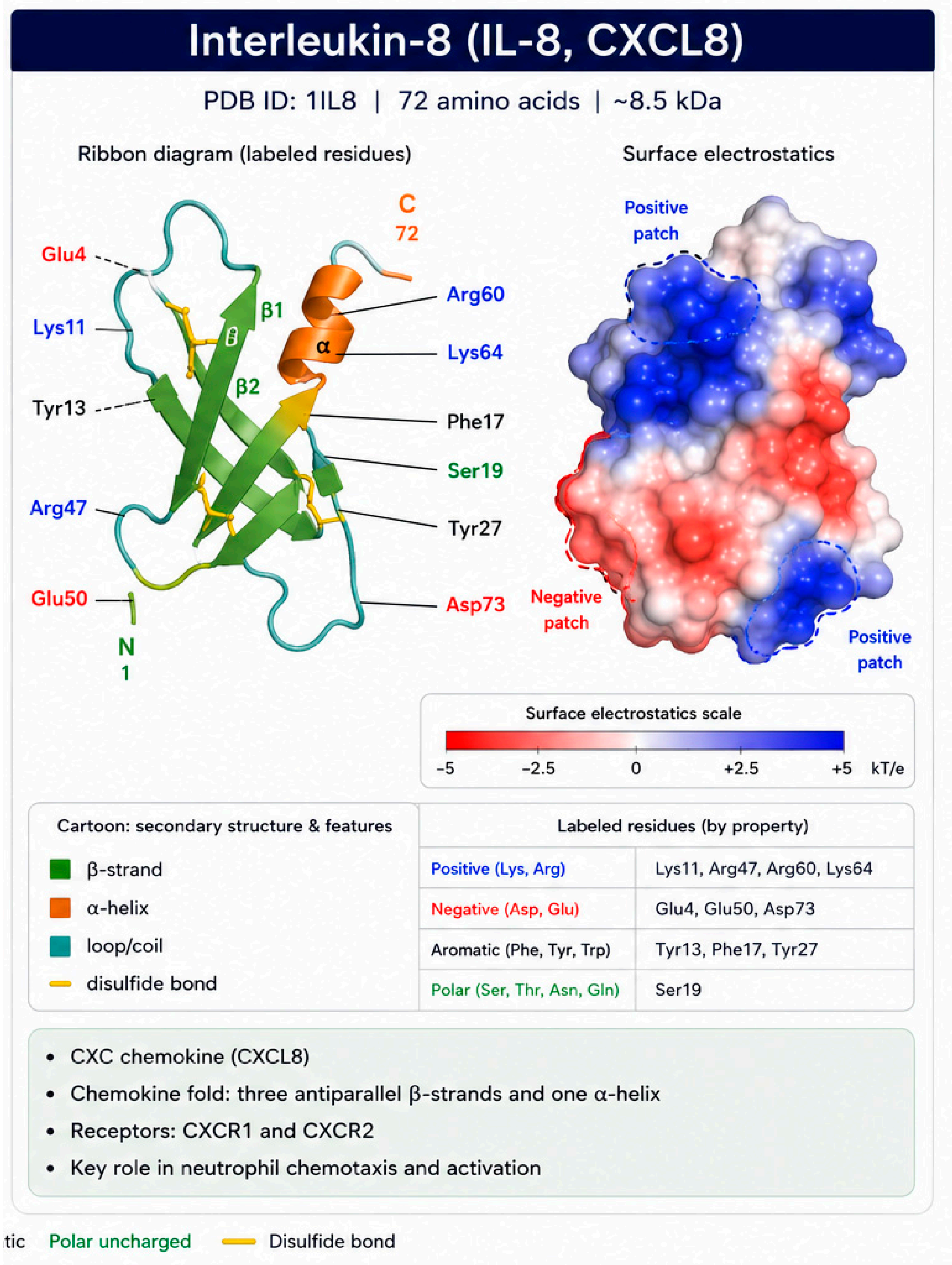

Figure S5. Structural representation of human interleukin-8 (IL-8). Ribbon and electrostatic surface representations with schematic annotation of representative amino acid residues. Created by the authors based on PDB structure 1IL8.

Describing a molecule through its amino acid composition does not explain the differences in the energy of interaction between individual sections of the protein and the energetically heterogeneous surface of the carbon material.

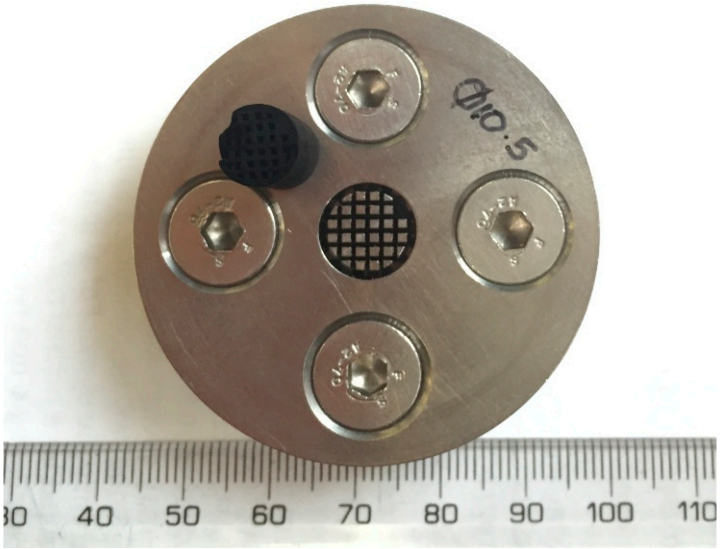

Figure S6. Describes the principle of die extrusion and shows a diagram of the unit cell of the die for extruding honeycomb monoliths.

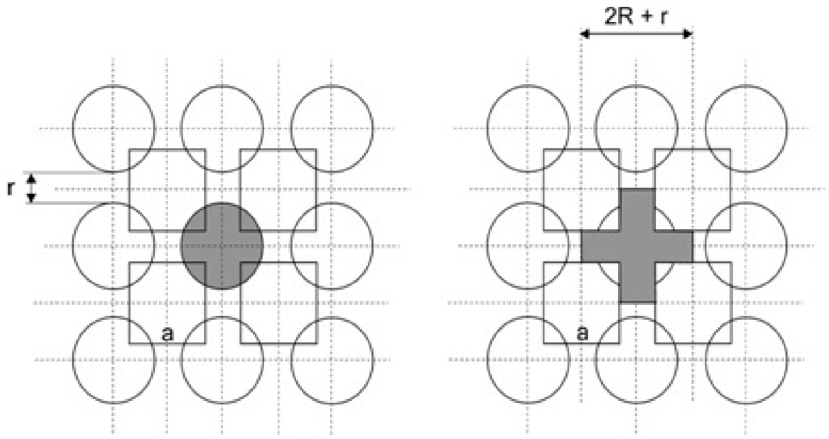

A cylinder is pressed through holes of area Pi*R^2.

At the outlet of the die, the cylindrical volume is redistributed into a volume with a cross-section of area $(2R + r)^2 - a^2$.

The volume of mass entering through the opening must be equal to the volume exiting through the crosspiece. Assuming equal heights, the requirement of equal volumes translates into equal areas:

$$(2R + r)^2 - a^2 = Pi*R^2$$

Figure S7. Schematic of the working section of the die for extruding honeycomb monoliths.

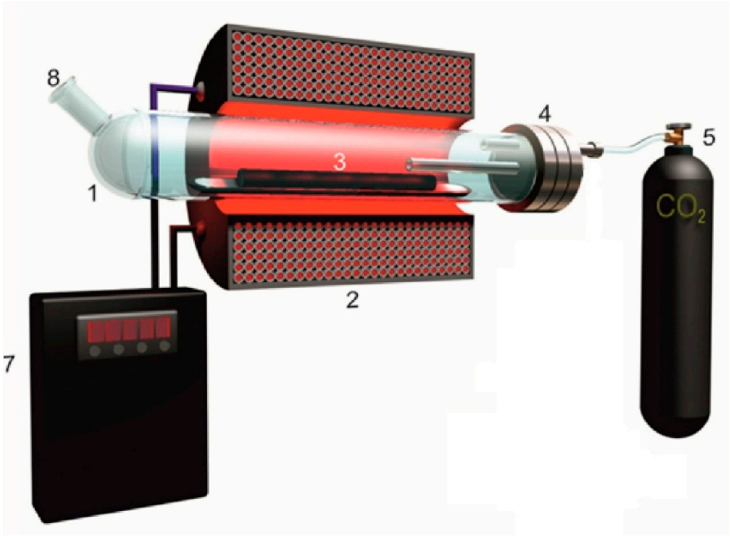

Figure S8. Schematic diagram of the carbonization and CO_2-gas activation system for carbon honeycomb monoliths. 1—Quartz reactor; 2—Electric furnace; 3—Boat with honeycomb monoliths; 4—Gas inlet;5—Steam generator; 6—Gas cylinder; 7—Temperature controller; 8—Exhaust gas outlet.

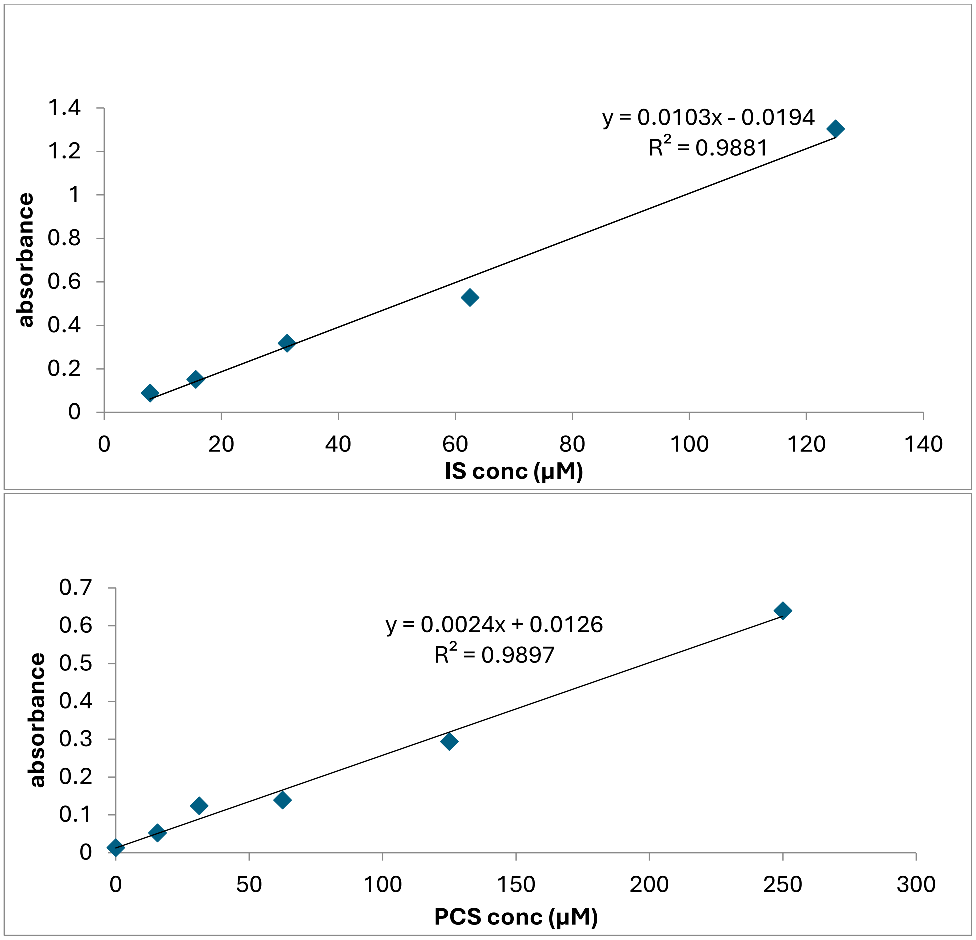

Figure S9. Standard curves for calculation of IS and PCS concentrations.

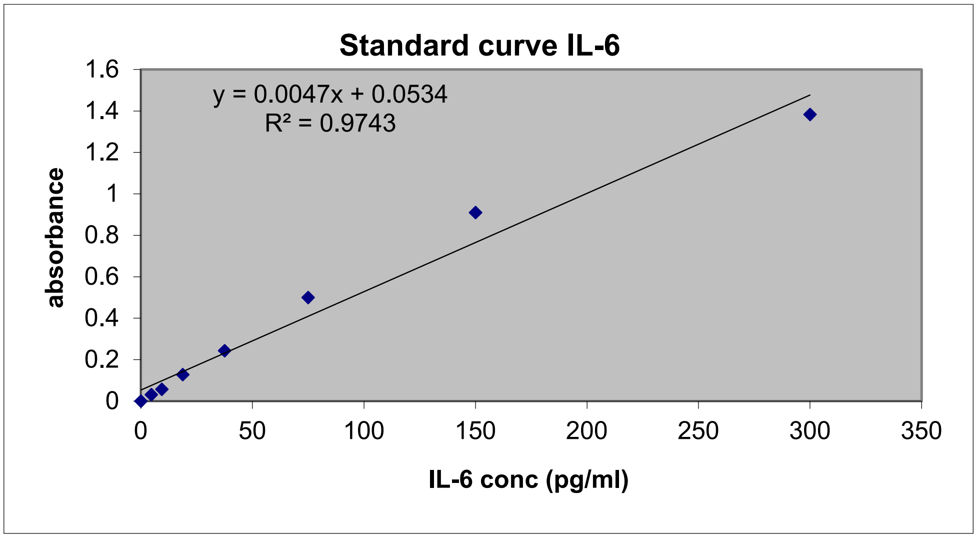

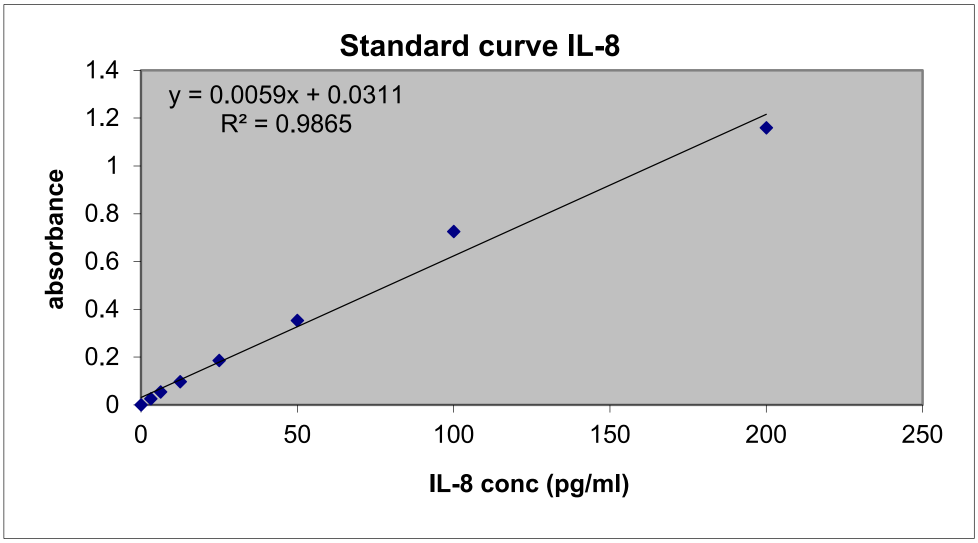

Figure S10. Standard curves for IL-6 and IL-8 calculation of concentrations.
